# Thioredoxin A Is Essential for Motility and Contributes to Host Infection of *Listeria monocytogenes* via Redox Interactions

**DOI:** 10.3389/fcimb.2017.00287

**Published:** 2017-06-28

**Authors:** Changyong Cheng, Zhimei Dong, Xiao Han, Hang Wang, Li Jiang, Jing Sun, Yongchun Yang, Tiantian Ma, Chunyan Shao, Xiaodu Wang, Zhongwei Chen, Weihuan Fang, Nancy E. Freitag, Huarong Huang, Houhui Song

**Affiliations:** ^1^China-Australian Joint Laboratory for Animal Health Big Data Analytics, Zhejiang Provincial Engineering Laboratory for Animal Health Inspection and Internet Technology, College of Animal Science and Technology of Zhejiang A&F UniversityLin'an, China; ^2^Zhejiang University Institute of Preventive Veterinary Medicine and Zhejiang Provincial Key Laboratory of Preventive Veterinary MedicineHangzhou, China; ^3^Department of Microbiology and Immunology, University of Illinois at ChicagoChicago, IL, United States; ^4^Institute of Developmental and Regenerative Biology, College of Biological and Environmental Science, Hangzhou Normal UniversityZhejiang, China

**Keywords:** *Listeria monocytogenes*, thioredoxins, motility, host infection, redox interaction

## Abstract

Microbes employ the thioredoxin system to defend against oxidative stress and ensure correct disulfide bonding to maintain protein function. *Listeria monocytogenes* has been shown to encode a putative thioredoxin, TrxA, but its biological roles and underlying mechanisms remain unknown. Here, we showed that expression of *L. monocytogenes* TrxA is significantly induced in bacteria treated with the thiol-specific oxidizing agent, diamide. Deletion of *trxA* markedly compromised tolerance of the pathogen to diamide, and mainly impaired early stages of infection in human intestinal epithelial Caco-2 cells. In addition, most *trxA* mutant bacteria were not associated with polymerized actin, and the rare bacteria that were associated with polymerized actin displayed very short tails or clouds during infection. Deletion or constitutive overexpression of TrxA, which was regulated by SigH, severely attenuated the virulence of the pathogen. Transcriptome analysis of *L. monocytogenes* revealed over 270 genes that were differentially transcribed in the Δ*trxA* mutant compared to the wild-type, especially for the virulence-associated genes *plcA, mpl, hly, actA*, and *plcB*. Particularly, deletion of TrxA completely reduced LLO expression, and thereby led to a thoroughly impaired hemolytic activity. Expression of these virulence factors are positively regulated by the master regulator PrfA that was found here to use TrxA to maintain its reduced forms for activation. Interestingly, the *trxA* deletion mutant completely lacked flagella and was non-motile. We further confirmed that this deficiency is attributable to TrxA in maintaining the reduced intracellular monomer status of MogR, the key regulator for flagellar formation, to ensure correct dimerization. In summary, we demonstrated for the first time that *L. monocytogenes* thioredoxin A as a vital cellular reductase is essential for maintaining a highly reducing environment in the bacterial cytosol, which provides a favorable condition for protein folding and activation, and therefore contributes to bacterial virulence and motility.

## Introduction

*Listeria monocytogenes* is a Gram-positive foodborne pathogen that causes listeriosis leading to high mortality, especially in the aging population, infants, and immunocompromised individuals (Vazquez-Boland et al., 2001; Corr and O'Neill, 2009). The organism is well-adapted to various physiological environments, employing different strategies to counteract hostile acidity, osmolality, oxygen tension, and other stress conditions (Cotter et al., 2001; Wemekamp-Kamphuis et al., 2004; Cheng et al., 2013) present in the environment and within the vacuolar compartment of phagocytic cells (Kim et al., 2007). Among these, oxidative stress is an imbalance in electrons that can damage DNA, iron-sulfur clusters, lipids, and proteins. Oxidative stress is both produced by the bacteria (endogenous) and encountered in the environment (exogenous; Imlay, 2003). Therefore, bacteria have evolved diverse detoxification mechanisms to survive in oxygen-rich environments, including production of antioxidants and enzymes that consume damaging reactive oxygen species (ROS; Whiteley et al., 2017). The intracellular life cycle of *L. monocytogenes* begins when the bacterium is phagocytosed by a host cell, where it transiently resides within the oxidizing environment of the phagosome. The bacteria then secrete the pore-forming toxin listeriolysin O to escape from the phagosome and enter into the cytosol that is a highly reducing environment (Schnupf and Portnoy, 2007). The rapid transit from the oxidizing phagosome to the reducing cytosol, making *L. monocytogenes* an ideal model for studying adaptive responses to redox changes during infection.

The thioredoxin (Trx) system comprising nicotinamide adenine dinucleotide phosphate (NADP), thioredoxin and the partner enzyme, thioredoxin reductase (TrxR), is widely distributed in several species, from archaea and bacteria to human. This antioxidant system transfers electrons from NADP through TrxR to Trx and then to a large range of proteins that play critical roles in DNA synthesis and oxidative stress (Holmgren, 1985; McCarver and Lessner, 2014). The oxidoreductase, thioredoxin, contains a common structural fold (Trx domain) and is involved in cellular defense against oxidative stress caused by reactive oxygen species (ROS), such as hydrogen peroxide, hydroxyl radicals, and superoxide anions (Boles and Singh, 2008; Yoshioka et al., 2012). Excessive amounts of these highly reactive compounds may damage DNA, proteins, and lipid membranes (Sampayo et al., 2003). In the microbial system, recovery from ROS stress is accomplished by Trx and TrxR (Yoshida et al., 2003). In several bacterial species, such as *Staphylococcus aureus* (Uziel et al., 2004), *Escherichia coli* (Ritz et al., 2000), *Bacillus subtilis* (Leichert et al., 2003), *Rhodobacter sphaeroides* (Li et al., 2003), and *Brevibacillus choshinensis* (Tanaka et al., 2004), thioredoxins play a major role in the protection of cells against ROS as well as maintaining the intracellular redox homeostasis. Importantly, the Trx system is the major cellular disulfide reductase in cells, which provides the electron donor for many critical enzymes, including ribonucleotide reductase, Prx, and methionine sulfoxide reductase, and is thus involved in a range of cellular functions (Lu and Holmgren, 2014b). The host cytosol is a highly reducing environment and upon entry into this compartment, all thiols are expected to be in the reduced form, which provides a favorable environment for protein folding and activation. A good example is for activation of PrfA, the master regulator controlling expression of most virulence genes in *L. monocytogenes* (Reniere et al., 2015). It was recently suggested that reduced glutathione (GSH) can function as an essential small molecule cofactor of PrfA through allosteric binding to the protein, thereby increasing the activity of PrfA at target genes(Hall et al., 2016). Moreover, PrfA thiols can be reversibly oxidized by the intracellular reactive oxygen and nitrogen species, temporarily inactivating the protein by inhibiting DNA binding, and leading to a downregulation of PrfA-regulated genes (Reniere et al., 2015). Therefore, we hypothesized that *L. monocytogenes* TrxA, as a reductase, might be highly involved in maintaining redox homeostasis in the bacterium for PrfA activation, and thus contribute to bacterial infection.

In addition, for oxidative protein folding in *E. coli*, Trx donates electrons to the disulfide bond (DSB) system and contributes to isomerization of incorrectly paired disulfide bonds (Rozhkova et al., 2004). Since many bacterial virulence factors require stable disulfide bonds for proper folding and function, studies on oxidoreductases are important for understanding the mechanisms underlying bacterial pathogenesis and the potential development of novel therapeutics. The disulfide bond formation (DSB) system in *E. coli* that catalyzes the oxidative folding step consists of several enzymes that form two distinct pathways: oxidative and isomerization (Ito and Inaba, 2008). The Trx domain-containing oxidase, DsbA, is responsible for the introduction of disulfide bonds into substrate proteins and subsequently obtaining the oxidizing equivalent from the membrane protein, DsbB, the second protein in the oxidative folding pathway (Missiakas et al., 1993). Due to DsbA misoxidation or external oxidative stress, isomerization of incorrectly paired disulfides in *E. coli* is performed by the DsbC-DsbD system. Mispaired disulfide bonds accept electrons from the homodimeric periplasmic protein, DsbC. DsbC accepts electrons from the membrane protein, DsbD, which, in turn, accepts electrons from the cytoplasmic Trx system (Missiakas et al., 1994, 1995). A DsbC homolog, DsbG, also located in the *E. coli* periplasm, is reported to function as a disulfide isomerase (Bessette et al., 1999).

Interestingly, many Gram-positive bacteria have different complements of DSB proteins. Searches for orthologs of *B. subtilis* DsbA and DsbB (known as BdbD and BdbC, respectively) in low GC Gram-positive bacteria revealed that many of these organisms contain either a DsbA or a DsbB protein, but not both (Kouwen et al., 2007). *L. monocytogenes* encodes two putative DSB proteins, Lmo0964 and Lmo1059, annotated as DsbA-like and DsbG in the GenBank database, respectively. Lmo0964 was recently designated as a putative thioredoxin similar to YjbH in *Bacillus subtilis* and shown to contribute to expression of the ActA protein, required for *L. monocytogenes* actin based motility (Reniere et al., 2016). Moreover, homologs of the Trx system-related genes have been identified in the sequenced genome of *L. monocytogenes* EGD-e via *in silico* analysis (Glaser et al., 2001). Based on a pattern search for the CXXC motif, the characteristic structure of Trx in the EGD-e genome, six thioredoxin family proteins (Lmo1233, 1609, 1903, 2152, 2424, 2830) and one TrxR (Lmo2478), were considered likely candidates (Gopal et al., 2009). Only the modeled structure of Lmo1233 (TrxA) coincided with the canonical conformation of thioredoxin containing the highly conserved CGPC motif. However, none of the components of the Trx system from *L. monocytogenes* have been characterized to date. In the current study, we aimed to fully elucidate the molecular functions and underlying mechanisms of the *L. monocytogenes* Trx system, with a view to determining whether it contributes to biological processes related to bacterial survival and infection. Our collective data indicate that *L. monocytogenes* thioredoxin A contributes to bacterial virulence and motility through redox interactions. The results from this study provide a valuable model for clarifying the pathways associated with the potential roles of thioredoxins from foodborne pathogens in improving survival in the external environment, and more importantly, successfully establishing infection within the host.

## Results

### Cys28 and Cys31 are required for the oxidoreductase activity of TrxA

**Figure 1:**
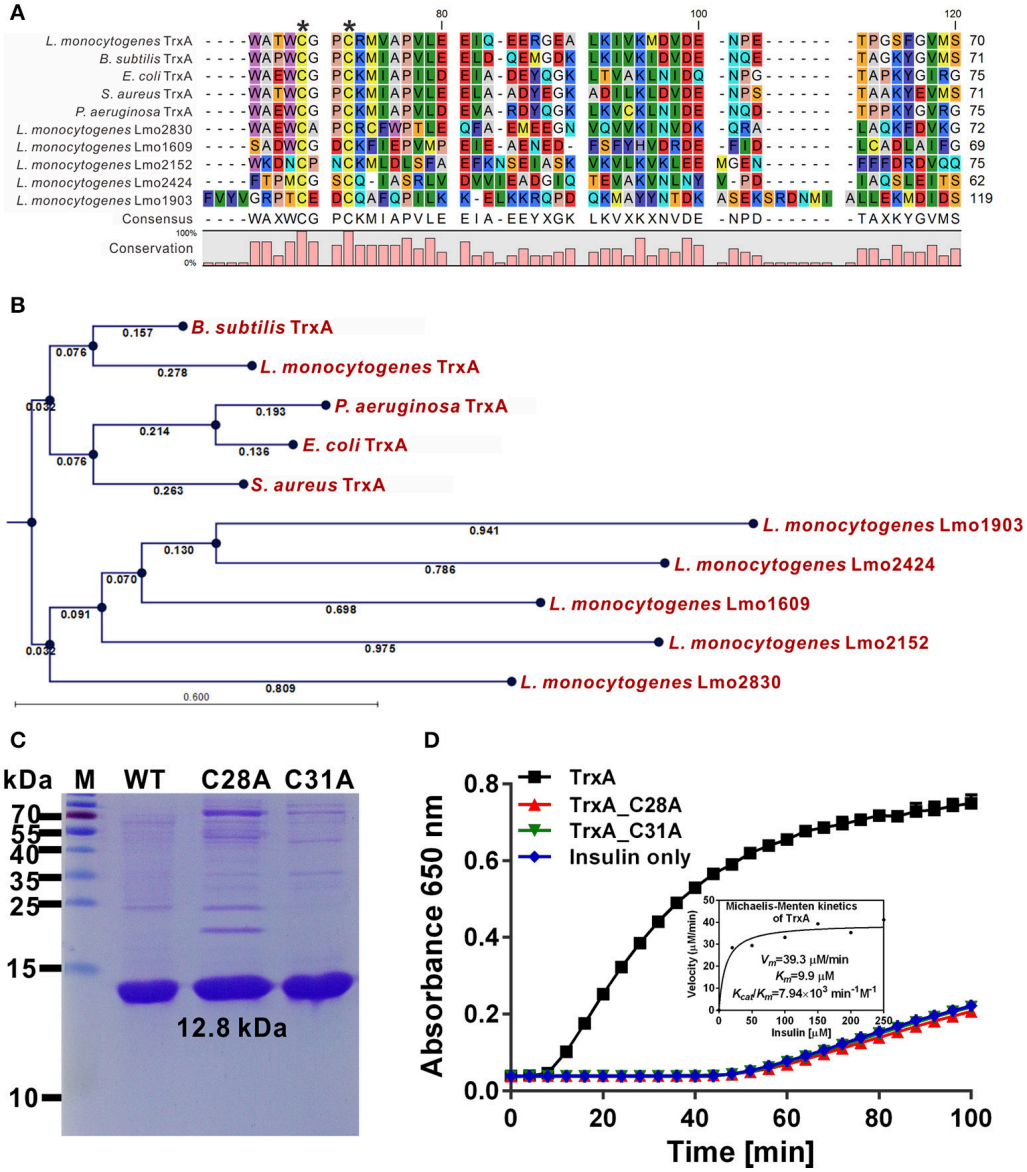
Cys28 and Cys31 of TrxA are required for oxidoreductase activity. **(A)** Amino acid sequence alignment of *L. monocytogenes* putative thioredoxins (TrxA, Lmo1609, 1903, 2152, 2424, and 2830) against homologs from *B. subtilis*, *E. coli*, *P. aeruginosa*, and *S*. *aureus*. Key amino acid residues denoted with asterisks are involved in catalytic activity. **(B)** Phylogenetic tree of *L. monocytogenes* putative thioredoxins and homologs from other bacterial species. The tree was constructed with the Neighbor-Joining (NJ) program and a bootstrap test of 100 replicates used to estimate the confidence of branching patterns, where the numbers on internal nodes represent the support values. **(C)** SDS-PAGE (12% gel) analysis of recombinant TrxA and its mutants, C28A and C31A. **(D)** *In vitro* insulin reduction assay of recombinant TrxA and its mutants, C28A and C31A. The inset shows the Michaelis-Menten plot and kinetic parameters, *K*_*m*_, *V*_*max*_, and *k*_*cat*_, for TrxA. Data are representative of three independent experiments.

Six putative homologs, Lmo1233, 1609, 1903, 2152, 2424, and 2830, were annotated as thioredoxin-like proteins (Gopal et al., 2009). Among these, only TrxA contained the canonical CGPC active motif of the thioredoxins (Figure 1A). Moreover, *L. monocytogenes* TrxA shows 40.0–64.1% amino acid sequence homology to the Trxs from other microorganisms (Figure 1A). Phylogenetic analysis further revealed TrxA and its homolog from *B. subtilis* form a sister branch, while the other five thioredoxin homologures of *L. monocytogenes* form a separate clade (Figure 1B), indicating that these Trxs within this clade are distinct from the TrxA found in other bacteria species. Recombinant TrxA efficiently catalyzed the thiol-disulfide oxidoreduction of insulin in the presence of DTT as an electron donor (Figures 1C,D). Both C28A and C31A single mutations completely abolished the oxidoreductase ability of the protein to catalyze the reduction of insulin (Figure 1D), clearly indicating that *L. monocytogenes trxA* encodes a functional thioredoxin and the residues Cys28 and Cys31 are required for its catalytic activity.

### TrxA is responsible for bacterial resistance to thiol-specific oxidative stress

**Figure 2:**
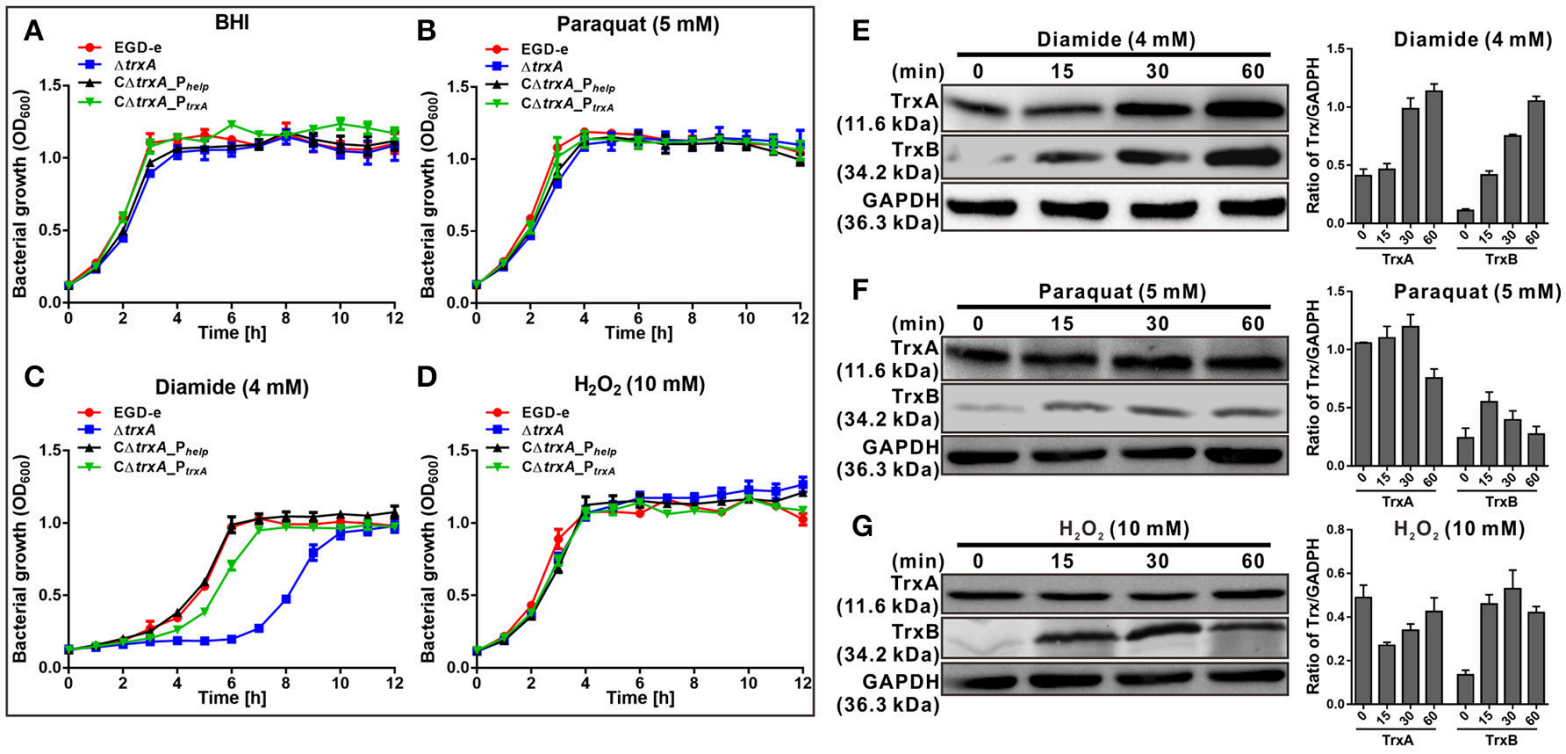
TrxA is responsible for bacterial resistance to thiol-specific oxidative stress. **(A–D)** Growth of *L. monocytogenes* wild-type EGD-e, mutant Δ*trxA* and its two complement strains, CΔ*trxA*_P_*help*_ and CΔ*trxA*_P_*trxA*_, in BHI broth supplemented with or without oxidative agents. Overnight bacteria were re-suspended in fresh BHI alone **(A)** or BHI supplemented with 5 mM paraquat **(B)**, 4 mM diamide **(C)** or 10 mM H_2_O_2_ **(D)**, and incubated at 37°C for 12 h. Kinetic growth at OD_600nm_ was measured at 1 h intervals. Data are expressed as means ± *SD* of three independent experiments. **(E–G)** Western blot analysis of TrxA expression under oxidative conditions. Total bacterial cell-free proteins were isolated at the indicated times after treatment with 4 mM diamide **(E)**, 4 mM paraquat **(F)** or 10 mM H_2_O_2_ **(G)**, and protein levels of TrxA and TrxB determined via western blot. GAPDH was used as the internal control. Results are indicated as a grayscale ratio of TrxA to GADPH or TrxB to GADPH. Data are expressed as means ± SD of three independent experiments.

Previous work suggested a connection between the *L. monocytogenes* redox response and virulence (Reniere et al., 2016). These observations prompted us to investigate the role of the thioredoxin in *L. monocytogenes*. The *trxA* mutant strain exhibited similar growth to the parent strain in normal BHI medium (Figure 2A). To further determine whether the Trx system could contribute to oxidative tolerance, the bacteria were exposed to different oxidative agents. For oxidative stresses, paraquat was used as a superoxide generating compound, and H_2_O_2_ as a direct oxidant while diamide was used as a thiol-specific oxidizing agent. Notably, deletion of *trxA* led to a significantly increased lag phase in growth, starting from 4 h (*P* < 0.01), compared to its parental strain and two complement strains (CΔ*trxA*_P_*trxA*_, expressing TrxA under its native promoter, and CΔ*trxA*_P_*help*_, carrying the constitutive *Listeria* promoter, P_*help*_) in BHI containing diamide, while H_2_O_2_ and paraquat did not exert marked effects on growth (Figures 2B–D). Immuno-blotting was used to determine expression of the Trx system under different oxidative stress conditions. As expected, diamide at a concentration of 4 mM induced expression of TrxA and TrxB (annotated as a thioredoxin reductase) to a significant extent, especially at the 30 and 60 min time-points (Figure 2E). However, no obvious induction was found in the presence of paraquat or H_2_O_2_ stress on TrxA expression, while TrxB was slightly induced by these two oxidants (Figures 2F,G). The results collectively suggest that diamide is a stronger inducer of TrxA expression than other oxidants, which enables *L. monocytogenes* to better survive in the thiol-specific oxidative conditions.

### The *L. monocytogenes* trxA gene deletion mutant lacks flagella and is non-motile

**Figure 3:**
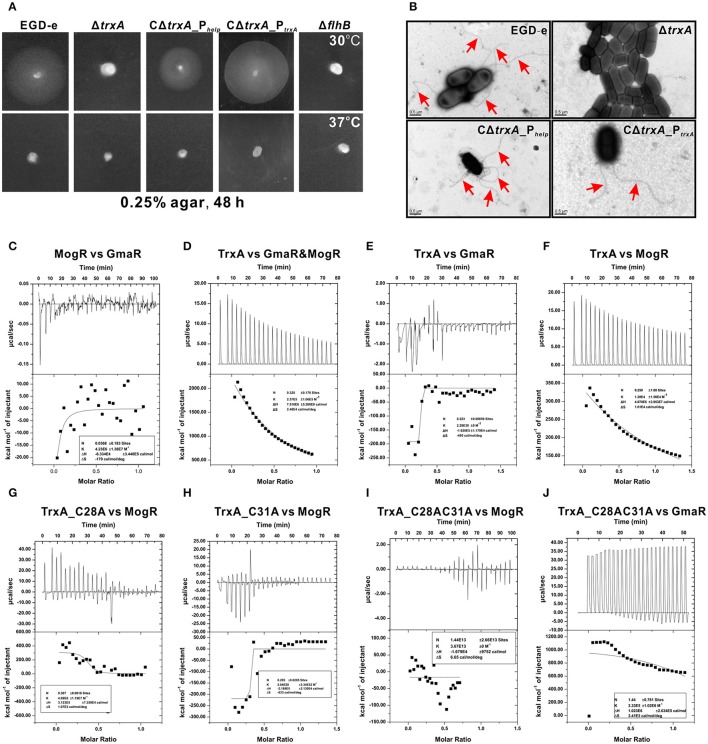
TrxA contributes to *L. monocytogenes* motility and flagellar formation *via* interactions with MogR and GmaR **(A,B)**. Motility assay **(A)** and transmission electron microscopy (TEM) **(B)** were performed on *L. monocytogenes* wild-type EGD-e, mutant Δ*trxA* and its two complement strains, CΔ*trxA*_P_*help*_ and CΔ*trxA*_P_*trxA*_, grown on soft agar (0.25%) at 30° or 37°C. Mutant Δ*flhB*, which completely abolished bacterial motility, was taken as a reference control. **(C–J)** Isothermal titration calorimetry (ITC) measurement of protein-protein interactions. **(C)** ITC of interactions between MogR, the flagellar gene transcriptional repressor, and the anti-repressor, GmaR. **(D)** ITC of TrxA with MogR and GmaR. **(E)** ITC of TrxA binding to GmaR. **(F)** ITC of TrxA interactions with MogR. **(G)** ITC of interactions between the single mutant TrxA (C28A) and MogR. **(H)** ITC of interactions between the single mutant TrxA (C31A) and MogR. **(I)** ITC of interactions between the double Cys28/Cys31 mutant of TrxA (C28AC31A) and MogR. **(J)** ITC of interactions between the double Cys28/Cys31 mutant of TrxA (C28AC31A) and GmaR. For all titrations, raw data are presented in the upper panel and fitted binding curves in the lower panel.

DsbA, a disulfide bond forming protein A comprising a Trx domain, catalyses the formation of disulfide bond that is involved in bacterial flagella synthesis (Heras et al., 2009). Thus, we examined whether TrxA is linked to motility in *Listeria*. Interestingly, motility of the Δ*trxA* strain at 30°C was almost completely compromised, compared to its wild-type counterpart (Figure 3A). Impaired motility of the mutant was fully rescued in the complement strain, CΔ*trxA*_P_*trxA*_, expressing TrxA under its native promoter, and partly restored in the other complement strain, CΔ*trxA*_P_*help*_, carrying the constitutive *Listeria* promoter, P_*help*_ (Figure 3A), suggesting that *L. monocytogenes* TrxA contributes to bacterial motility. In contrast, no motility was observed at 37°C (Figure 3A), which inhibited flagellar formation in many *Listeria* strains (Grundling et al., 2004). Transmission electron microscopy further revealed that the *trxA* mutant is totally unable to produce visible flagella whereas morphologically normal-looking flagella were expressed by the wild-type and two *trxA* complement strains (Figure 3B). Our results demonstrate for the first time that *L. monocytogenes* TrxA participates in bacterial motility and flagellar production.

### TrxA is involved in the interactions between MogR and GmaR

Temperature-dependent expression of *Listeria* flagellar motility genes is mediated by the opposing activities of MogR, a DNA-binding transcriptional repressor, and GmaR, an anti-repressor that functions as a direct antagonist of MogR (Shen and Higgins, 2006; Shen et al., 2006; Kamp and Higgins, 2011). Given that MogR and GmaR contain 2 and 4 cysteines, respectively, the CGPC domain of TrxA is predicted to play a critical role in the interactions between MogR and GmaR, and therefore contribute to *Listeria* flagella formation. Calorimetric response data on MogR during reaction with GmaR showed significant scatter with no discernible trends, which was more evident in the corresponding binding isotherm plot (Figure 3C), indicating that *in vitro* interactions between MogR and GmaR are not strong. However, upon titration of purified TrxA into MogR and GmaR, the binding event exhibited endothermic heat of reaction. A good fit to the data for calorimetric titration was achieved using a single-site binding model (Figure 3D), suggesting that TrxA is required to increase the binding affinity of MogR to GmaR. TrxA displayed endothermic reactions for strong binding to MogR in a manner similar to calorimetric data obtained in Figure 3D, while showing weak binding affinity to GmaR (Figures 3E,F). Notably, the endothermic response clearly observed during the whole titration process between TrxA and MogR is believed to arise due to breakage of intermolecular bonds among MogR molecules as a consequence of titration of TrxA (Figure 3F), suggesting that this protein contributes to breaking the intermolecular bonds of MogR that bind DNA of the flagella-related gene promoter region as a dimer (Shen et al., 2009). More importantly, mutation of either C28A or C31A located within the critical motif of thioredoxin significantly weakened the binding affinity of TrxA to MogR and GmaR, as determined from calorimetric data (Figures 3G–J).

### DSB oxidative and isomerization pathways are required for protein folding

**Figure 4:**
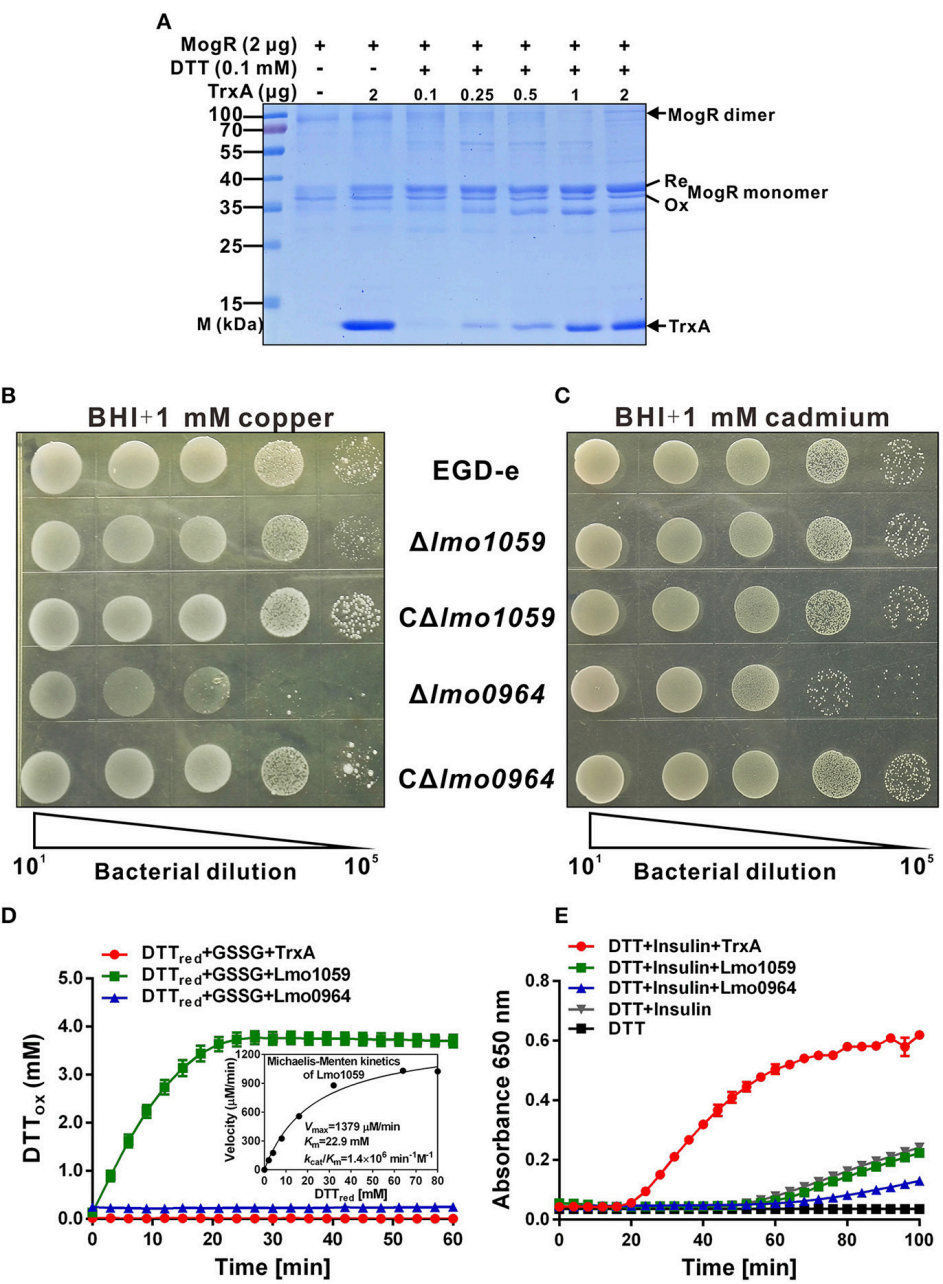
The redox state of MogR and enzymatic analysis and metal sensitivity assay. **(A)** The *in vitro* redox state of MogR changes under the efficient reduction by TrxA. MogR is reduced following incubation with the increasing amount of TrxA in the presence of DTT, and the redox state of MogR was visualized by SDS-PAGE and Coomassie blue staining. The reduced and oxidized forms of MogR are indicated as Re and Ox, respectively. **(B,C)** *L. monocytogenes* WT EGD-e, Δ*lmo1059*, Δ*lmo0964* and the complement strains, CΔ*lmo1059* and CΔ*lmo0964*, were subjected to copper **(A)** and cadmium **(B)** sensitivity assays by spotting 10^7^ cfu of each bacterial strain onto BHI plates containing 1 mM copper and 1 mM cadmium. **(D)** The disulfide interchange reaction was used to determine the isomerase activities of TrxA, Lmo1059, and Lmo0964. The inset depicts the Michaelis-Menten plot and kinetic parameters, *K*_*m*_, *V*_*max*_, and *k*_*cat*_, for Lmo1059. **(E)** *In vitro* insulin reductase activity was assayed for recombinant TrxA, Lmo1059, and Lmo0964 over a period of 100 min. Data in **(D,E)** are expressed as means ± SD of three independent experiments.

As indicated from isothermal titration calorimetry (ITC) data (Figure 3), TrxA could contribute to catalyzing the reduction of misfolded MogR disulfides and maintaining its intracellular monomer status. To further characterize the reduction properties of recombinant TrxA, we assayed the ability of the thioredoxin to reduce MogR *in vitro*. The thioredoxin assay directly measures the redox state of the substrate by using a gel-based assay. In the presence of SDS, a protein with free cysteine residues is less compact than the same protein in which these residues form disulfide bonds. The reduced form of a protein therefore migrates more slowly upon polyacrylamide gel electrophoresis (PAGE) in the presence of SDS than its oxidized counterpart. As indicated in the Figure 4A, the recombinant MogR monomer exists as a mixture of the reduced and oxidized forms. However, the reduced form amount of MogR monomers gradually increased when an increasing amount of the recombinant TrxA added in the presence of DTT as an electron donor, while the amount of MogR dimers decreased (Figure 4A), strongly confirming that TrxA could act to reduce disulfides in MogR and maintain the monomer status.

In addition, the thioredoxin system is the major cellular disulfide reductase in cells, which can facilitate correct oxidative protein folding together with protein disulfide isomerases (PDI) and DSB proteins (Lu and Holmgren, 2014b). Searches for orthologs of *B. subtilis* DsbA and DsbB in *L. monocytogenes* revealed that this pathogen only encodes two putative DSB proteins, DsbA (Lmo0964), and DsbG (Lmo1059), but does not encode DsbB or any other DSB proteins. Therefore, the roles of Lmo0964 and Lmo1059 in correct dimerization of MogR were further investigated. To investigate mutant sensitivity to oxidative stress, *Listeria* were grown in the presence of the redox-active metal, copper, which rapidly and randomly oxidizes unpaired cysteines through a superoxide mechanism (Hiniker et al., 2005). WT and Δ*lmo1059* had a similar resistance to copper while Δ*lmo0964* was extremely copper-sensitive (Figure 4B). Cadmium sensitivity was further tested as an indicator of oxidase capacity due to the high affinity of Cd^2+^ for protein thiol groups (Vallee and Ulmer, 1972). The *lmo0964*-null strain was especially Cd^2+^ sensitive, while wild-type and Δ*lmo1059* strains were resistant to a concentration of 1 mM Cd^2+^ (Figure 4C). Our results suggest that Lmo0964 acts not only as an oxidase but also an isomerase, whereas bacteria in the absence of *lmo1059* remain cadmium- and copper-resistant. Interestingly, *in vitro* enzymatic reactions indicate that Lmo1059 exhibits high disulfide interchange activity (Figure 4D) but fails to catalyze reduction of insulin in the presence of DTT (Figure 4E), suggesting that this protein mainly functions as an isomerase but not an oxidoreductase *in vitro*. Intriguingly, distinct from *E. coli* DsbA or BdbD in *B. subtilis*, Lmo0964 is unable to catalyze reduction of insulin, at least under standard assay conditions, suggesting that this protein is more stable in its oxidized form and mainly functions as an oxidase *in vitro*.

### TrxA alters global expression profiles of *L. monocytogenes* under oxidative conditions, especially expression of virulence-associated genes

**Figure 5:**
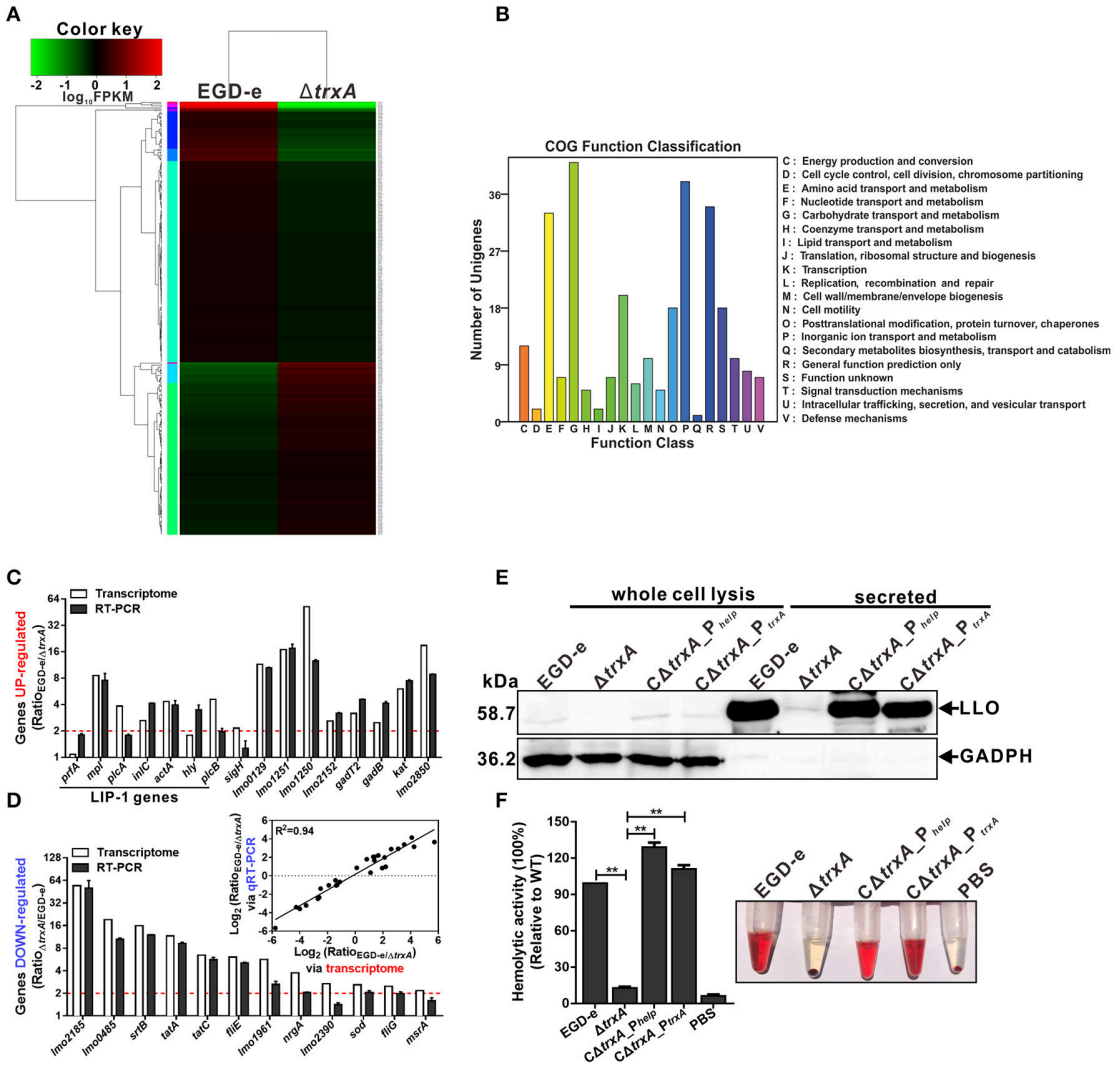
*L. monocytogenes* TrxA alters global gene expression profiles, especially regulating genes encoding virulence-associated factors. **(A)** Cluster analysis of differentially expressed genes in the WT and Δ*trxA* mutant strains via two-dimensional hierarchical clustering. The red color indicates upregulation, green indicates downregulation and black indicates no changes, compared to wild-type. The X-axis represents samples while the Y-axis represents genes. **(B)** Clusters of orthologous groups (COG) classification for differentially expressed unigenes in the WT and Δ*trxA* mutant strains. A total of 274 proteins were aligned to the COG protein database and classified functionally into 25 classes. Each function class is denoted by different capital letters under the x-axis. The y-axis represents the number of unigenes in a corresponding function class. **(C,D)** qRT-PCR validation of the transcriptome. Twenty-eight genes were randomly selected for qRT-PCR validation, including 16 representative upregulated genes **(C)** and 12 representative downregulated genes **(D)**. The inset shows normalized relationships between qRT-PCR and transcriptomes of randomly selected genes. Values are presented as the log_2_ ratio (EGD-e/Δ*trxA*) for genes. The coefficient of determination (*r*^2^) is indicated. All qRT-PCR data are expressed as means ± *SD* of three independent experiments. **(E,F)** TrxA is essential for LLO expression and hemolytic activity. Bacterial overnight cultures of *L. monocytogenes* strains were diluted into 100 mL fresh BHI and grown to the stationary phase. Bacterial pellets and cultures were collected to obtain the different cell fractions, as described in Section Materials and Methods. **(E)** Proteins were separated via 12% SDS-PAGE and immunoblotted with α-LLO or α-GAPDH antisera. The MW of each protein is indicated on the left. GAPDH was used as an internal control. **(F)** LLO-associated hemolytic activity was assessed by lysis of sheep red blood cells from serial dilutions of culture supernatants of bacterial strains. Data are expressed as means ± SD of three independent experiments. ^**^P < 0.01.

Whole-genome transcriptomic sequencing revealed that 160 genes show = 2.0-fold-higher transcript levels in parent EGD-e than the Δ*trxA* mutant strain under oxidative conditions (expo0sure of stationary cells to 2 mM diamide for 1 h; Table S1, Figures 5A,B). Transcript levels of a total of 114 genes were =2.0-fold higher in the Δ*trxA* mutant than the parent strain (Table S2, Figures 5A,B). Notably, the virulence genes, *plcA, mpl, actA*, and *plcB*, showed significantly higher expression in wild-type than the Δ*trxA* mutant, with transcriptional ratios consistently >3.8 (Table S1). The internalin gene, *inlC*, also showed significant expression ratios of >2.0 (Table S1). In addition, three other virulence-associated genes, *hly, inlA*, and *inlB*, were slightly activated by TrxA (Table S1). Transcriptomic data were validated using quantitative RT-PCR, showing that the results of transcriptome were firmly reliable (Figures 5C,D). In addition, deletion of TrxA almost completely reduced cytoplasm LLO expression and significantly inhibited its secretion into culture supernatant, compared to the wild-type and complement strains (Figure 5E), which was further verified by the finding that loss of TrxA leads to thoroughly reduced LLO activity in lysis of sheep red blood cells (Figure 5F). These results collectively show for the first time that *L. monocytogenes* TrxA alters global expression profiles, especially those of genes encoding virulence-associated factors, and therefore most probably play a critical role in bacterial pathogenicity.

### TrxA is required for early stages of *L. monocytogenes* infection and virulence within mice

**Figure 6:**
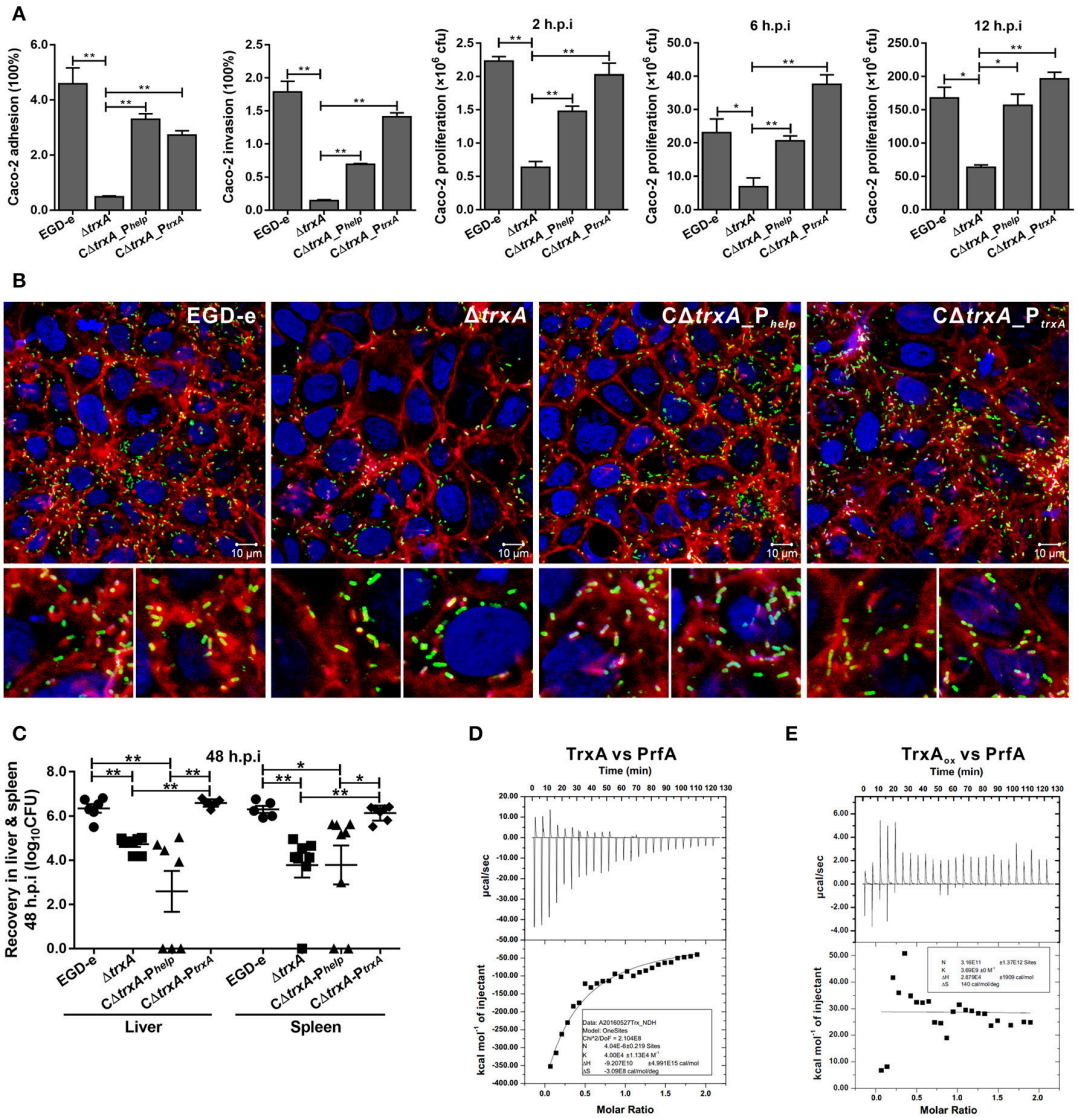
TrxA contributes to *L. monocytogenes* infection and virulence within mice. **(A)** Adhesion, invasion, and proliferation of *L. monocytogenes* in human intestinal epithelial cells, Caco-2. Cells infected with *L. monocytogenes* strains at the indicated time points were lysed and viable bacteria serially plated on BHI agar plates. The number of bacteria able to invade cells and survive are expressed as means ± SD of the recovery rate for each strain. ^*^P < 0.05; ^**^P < 0.01. **(B)** Actin tail formation in Caco-2 cells infected with *L. monocytogenes* strains 6 h post inoculation. Bacteria were detected with anti-Lm (green), bacteria actin tails and host actin using phalloidine (red), and cell nucleus labeled with DAPI (blue). Scale bar is 10 μm. The high-magnification images displayed at the bottom of each image show F-actin (red), bacteria (green), and nuclei (blue). **(C)** Proliferation of *L. monocytogenes* in mouse organs. Mice inoculated i.p. with *L. monocytogenes* strains (1 × 10^6^ CFU) were euthanized 48 h after infection, and organs (liver and spleen) recovered and homogenized. Homogenates were serially plated on BHI agar. Numbers of bacteria colonized in liver and spleen are expressed as means ± *SD* of the recovery rate per organ for each group. ^*^P < 0.05; ^**^P < 0.01; ns means no significance. **(D,E)** The interactions analysis of TrxA (D) or the oxidized forms of TrxA (TrxA_ox_) **(E)** with PrfA, the central virulence regulator of *Listeria*, by using the ITC assay. For all titrations, raw data are presented in the upper panel and fitted binding curves in the lower panel.

To investigate the involvement of TrxA in *L. monocytogenes* infection inside host cells, the epithelial cells Caco-2 was used to compare the wild-type and *trxA* mutant strains in terms of adhesion, invasion, and intracellular survival. The *trxA* mutant was significantly less adhesive and invasive, therefore resulting in the less efficient intracellular survival, compared to that of the wild-type strain (Figures 6A,B). Notably, EGD-e and the complement strains were able to associate with F-actin and formed long actin tails (Figure 6B), while a large proportion of the *trxA* mutant bacteria were not associated with polymerized actin, and the rare bacteria that were associated with polymerized actin displayed very short tails or clouds and were often located next to the plasma membrane (Figure 6B). However, unlike the circumstance in epithelial cells, the Δ*trxA* mutant did not show a significantly decreased ability to grow intracellularly in RAW264.7 macrophages relative to the wild-type strain (data not shown), suggesting that TrxA mainly contributes to the early stages of *L. monocytogenes* infection within host. Moreover, the Δ*trxA* mutant exhibited severely attenuated virulence, as infected mice exhibited bacterial burdens that were significantly lower relative to those infected with the wild-type strain at 48 h post infection (Figure 6C). Most importantly, the mutant strain containing the entire *trxA* CDS with its native promoter fully complemented virulence defects associated with loss of *trxA*, but not the P_*help*_ promoter (Figure 6C), suggesting no expression or overexpression of TrxA *in vivo* would disturb the equilibrium of the intracellular redox potential and thus bring harm to this pathogen for its ability to establish infections. Previous work suggested that the sigma factor H (SigH) is important for the regulation of mycobacterial response to oxidative stress by regulating the expression of thioredoxins (*trxB1* and *trxC*) and thioredoxin reductase (Bhat et al., 2012), which therefore prompted us to investigate the role of SigH in regulating on the Trx expression in *L. monocytogenes*. As determined by EMSA assay, the purified recombinant SigH bound to the fragment containing the promoter region of *trxA* or *trxB*. Besides, upon incubation of the promoter DNA fragment with increasing amounts of SigH, shifted, supershifted, and super-supershifted DNA complexes were observed (Figure S1), supporting the theory that the thioredoxin system of *L. monocytogenes* is strictly regulated by an alternative sigma factor. Taken together, these data demonstrate that TrxA-mediated regulation of virulence-associated factors constitutes an important aspect of *L. monocytogenes* infection within host. Importantly, the ITC experiment indicates that TrxA exhibits exothermic reactions for strong binding to the virulence regulator, PrfA, a member of the Crp/Fnr family of bacterial transcription factors (Figure 6D), while the oxidized forms of TrxA (TrxA_ox_) almost showed no binding affinity to PrfA (Figure 6E). Given the fact that PrfA activation is highly dependent on the reducing environment and only the reduced PrfA dimers can bind to DNA and then activate transcription of virulence genes (Reniere et al., 2015), we therefore suggest that TrxA plays a critical role in reducing the intermolecular disulfide bonds of PrfA and subsequently maintaining the intracellular monomer status for further dimerization.

## Discussion

*L. monocytogenes* adapts to a range of environmental extremes, including high salt concentrations, low pH, and various oxidative stress conditions in nature and host cells (Begley et al., 2010). Thioredoxins play major roles in protection of cells against toxic oxygen species as well as maintaining the intracellular thio-disulfide balance in bacteria (Uziel et al., 2004). In our experiments, *Listeria* depleted of *trxA* displayed significant growth defects upon exposure to diamide. Diamide, a thiol-specific oxidizing agent, induces glutathione depletion by trapping the free-SH group to increase the redox potential, and is therefore used to mimic oxidative shift in the cellular thiol-disulfide redox state (disulfide stress; Kallifidas et al., 2010). Cysteine thiols in bacterial proteins fulfill an important and diverse set of cellular functions, and ROS and reactive nitrogen species, so-called “reactive electrophilic species” (RES), affect the thiol redox balance (Pother et al., 2009). The toxicity of diamide is based on the formation of nonnative inter- and intra-molecular disulfide bonds that result in damage of proteins (Pother et al., 2009). We here found that the *Listeria* Trx system is differentially induced by a variety of agents that generate oxidative stress, implying that different regulatory mechanisms are involved in controlling the expression of *trxA* and other genes that participate in the oxidative stress response.

**Figure 7:**
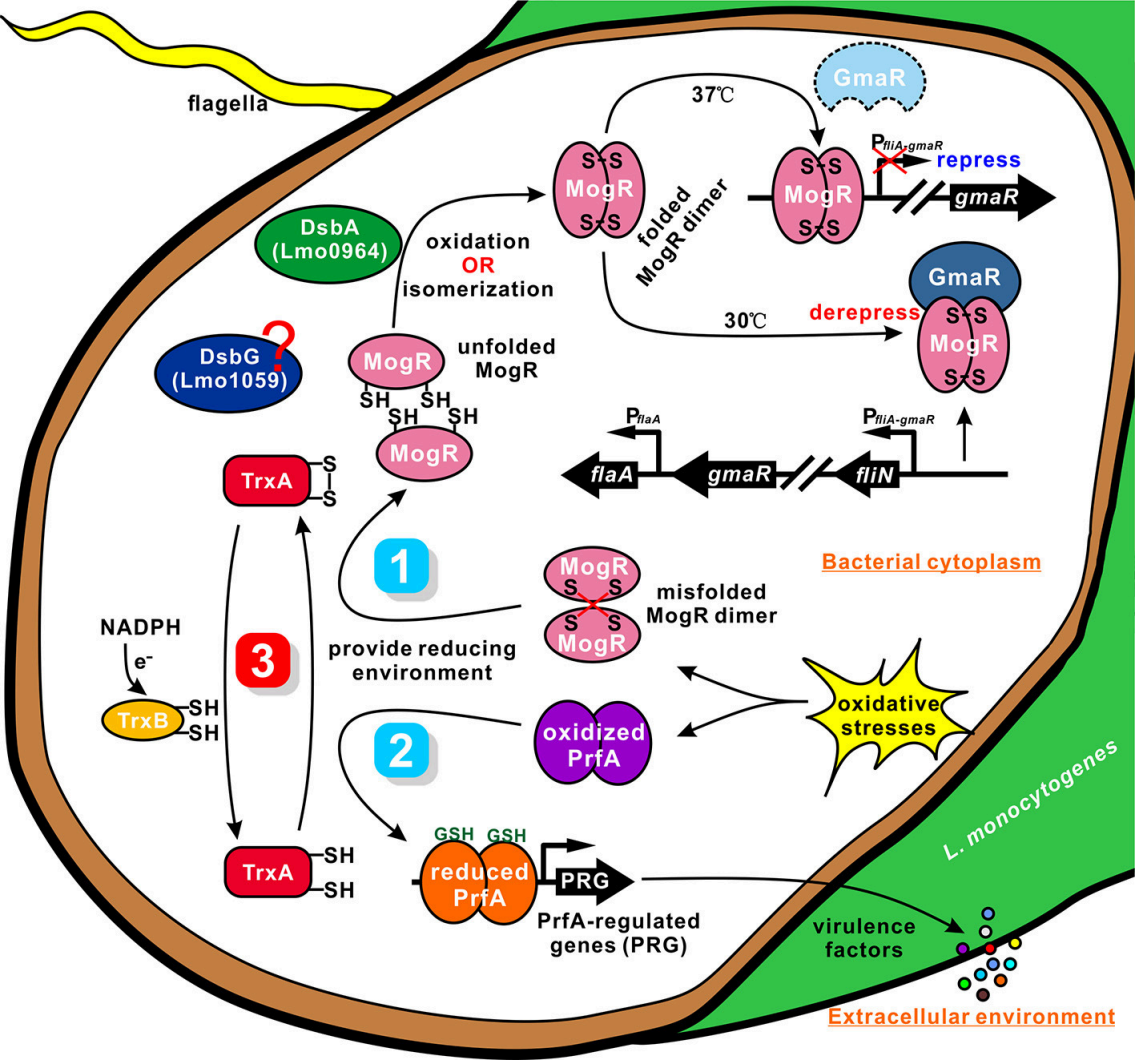
A proposed model for TrxA as a vital cellular reductase in *L. monocytogenes*. We propose that TrxA is essential for maintaining a highly reducing environment in the bacterial cytosol, which provides a favorable condition for protein folding and activation. Lmo0964 (DsbA) is responsible for direct oxidization of disulfide bonds in reduced MogR. Once MogR is correctly dimerized, the MogR:GmaR anti-repression complex is formed, which removes MogR from all flagellar motility gene promoters, enabling flagellar motility gene transcription at low temperatures. PrfA contains four cysteine residues in each monomer. TrxA contributes to maintain the reduced PrfA dimer that is exclusively activated in the cytosol in the presence of the co-factor GSH, and subsequently triggering expression of the virulence-associated factors.

The thioredoxin (Trx) system, which is composed of NADPH, thioredoxin reductase (TrxR), and thioredoxin, is a key antioxidant system in defense against oxidative stress through its disulfide reductase activity regulating protein dithiol/disulfide balance (Lu and Holmgren, 2014a; Shelar et al., 2015). Thioredoxins are proposed to participate directly in the oxidative stress response owing to their capability to reduce oxidized proteins. However, these proteins can also participate indirectly by affecting the expression of other genes involved in this response. Smits et al. (2005) previously reported the effects of thioredoxin depletion on global transcription levels in *B. subtilis*, whereby a total of 636 genes showed >2-fold changes in transcription levels. Effective depletion of thioredoxin A from *B. subtilis* induced genes involved in the oxidative stress response (but not those dependent on PerR), phage-related functions, and sulfur utilization. Moreover, several stationary phase processes, such as sporulation and competence, were affected. Since thioredoxins have not been reported to act as transcriptional regulators, the authors suggested that these transcriptional changes are likely to represent indirect effects of thioredoxin A (e.g., interactions with or influence on transcription factors or other proteins; Smits et al., 2005). Moreover, thioredoxins are involved in redox-dependent regulation of photosynthesis genes in *Rhodobacter* (Li et al., 2003). Importantly, in the current study, the known *Listeria* virulence genes, *hly, plcA, mpl, actA*, and *plcB*, showed significantly higher expression in wild-type than the Δ*trxA* mutant strain. These data imply that downregulation of expression of virulence factors in the mutant strain contributes significantly to the virulence defects. Bacteria express a range of disulfide-bonded virulence factors, including secreted toxins, surface components, such as adhesins and pili, and secretion systems. To ensure functional activity, many of these proteins must be oxidatively folded (Heras et al., 2009). The thioredoxin system is the major cellular disulfide reductase in cells, which can provide a highly reducing environment and then function as an effector to facilitate correct oxidative protein folding, together with protein disulfide isomerases (PDI) and DSB proteins (Lu and Holmgren, 2014b). PrfA is a member of the cAMP receptor protein (Crp) family of transcription factors, which are characterized by their allosteric regulation *via* small-molecule activators. In *L. monocytogenes*, PrfA is exclusively activated in the cytosol of host cells, leading to the assumption that the activating cofactor for PrfA is specific to this compartment (Reniere et al., 2015). Recently, glutathione (GSH), either generated by bacteria or derived from host cells, was found to be the essential small molecule cofactor of PrfA through allosteric binding to the protein (Hall et al., 2016). The process of infection or intercellular spread requires that *L. monocytogenes* inhabit an oxidizing vacuole, which may contain both reactive oxygen and nitrogen species. Upon oxidation, glutathione dimerizes to GSSG, which does not bind PrfA, owning to the ternary complex structure of PrfA cannot accommodate the larger oxidized glutathione (GSSG; Wang et al., 2017). In addition, PrfA thiols may be reversibly oxidized, temporarily inactivating the protein by inhibiting DNA binding and leading to a downregulation of PrfA-regulated genes (Reniere et al., 2015). It has been proposed that PrfA activation constitutes a two-step process requiring reduced protein thiols for initial DNA binding and allosteric binding of glutathione to PrfA for fully transcriptional activation (Reniere et al., 2015), indicating that the GSH-activated PrfA is primed for DNA binding (Hall et al., 2016). Consistent with this, the glutathione synthase (*gshF*)-deficient *L. monocytogenes* was moderately more sensitive to oxidative stress *in vitro*, expressed lower levels of the PrfA dependent virulence factors (ActA, LLO, and Hpt) in cells, formed very small plaques in tissue culture assay, and thus was attenuated *in vivo* (Reniere et al., 2015). Similarly, it was recently reported that LLO can be posttranslationally modified by S-glutathionylation at the conserved cysteine residue (Cys484) and that either endogenously synthesized or exogenously added glutathione was sufficient to form this modification. When recapitulated with purified protein *in vitro*, this modification completely ablated the activity of LLO, and this inhibitory effect was fully reversible by treatment with reducing agents (Portman et al., 2017). Previously, it has been showed that, GILT, a soluble thiol reductase expressed constitutively within the lysosomes of antigen-presenting cells, is able to activate LLO within the phagosome by the thiol reductase mechanism shared by members of the thioredoxin family, and therefore acts a critical host factor that facilitates *L. monocytogenes* infection (Singh et al., 2008). Here, we showed that TrxA exhibits exothermic reactions for strong binding to PrfA, suggesting the thioredoxin could contribute to provide a highly reducing environment for reduced PrfA maintaining, and therefore acts as an indirectly-activating factor (like GshF) of PrfA, thereby providing a reasonable explanation for our finding that deletion of *trxA* resulted in a significant downregulation of PrfA-regulated virulence genes, and exhibited less virulent in mice. Based on the findings, we conclude that TrxA modulates the expression of *Listeria* virulence factors, possibly through participating in the correct folding mechanism and activating of PrfA. A similar mechanism has been illustrated in *D. hafniense* CprK (also belonging to the Crp/Fnr family), whose activation requires not only a structural change induced by o-chlorophenol binding, but also a redox switch. The reduced CprK specifically binds to DNA. In addition, CprK can be reversibly inactivated by oxygen through formation of intermolecular Cys11–Cys200 disulfide bridges (Levy et al., 2008). Given that PrfA contains four cysteine residues in each monomer, we thus propose that TrxA could maintain redox homeostasis in the bacterium to reduce the intermolecular disulfide bonds of PrfA for further dimerization (Figure 7).

Many bacteria with mutations in either *dsbA* or *dsbB* are nonmotile owing to their inability to produce functional flagella (Dailey and Berg, 1993; Hayashi et al., 2000; Hiniker and Bardwell, 2004; Coulthurst et al., 2008). This defect is best characterized in *E. coli*, in which DsbA catalyzes the formation of an intramolecular disulfide bond between two cysteine residues in the flagellar P-ring motor protein, FlgI (Dailey and Berg, 1993). The lack of correctly folded FlgI may prevent subsequent integration of the major FliC component in the flagella organelle (Hiniker and Bardwell, 2004). TrxA displays endothermic reactions for strong binding to MogR, indicating that the protein contributes to breakage of the intermolecular bonds of MogR (containing two cysteines) that binds DNA of the flagella-related gene promoter as a dimer in *Listeria*. Accordingly, TrxA is speculated to play a critical role as a strong reductase in reducing the mismatched intermolecular disulfide bonds of MogR and maintaining the intracellular monomer status. Based on the findings of TrxA in PrfA activation, we proposed a similar mechanism for TrxA-dependent modification of MogR to ensure correct dimerization and flagellar gene synthesis (Figure 7). As an oxidoreductase, TrxA plays a critical role in reducing the mismatched intermolecular disulfide bonds of MogR and maintaining the intracellular monomer status for further dimerization. Moreover, Lmo0964 is a bifunctional protein that oxidizes and isomerizes disulfide bonds required for direct oxidization of reduced MogR to form disulfide bonds. Deletion of Lmo0964 markedly impaired *Listeria* motility while no significant differences in motility were observed between the Lmo1059-null mutant and wild-type (Figure S2), we suggest that the isomerase Lmo1059 might act as a backup system. Correct dimerization of MogR facilitates the formation of a MogR:GmaR anti-repression complex, which removes MogR from all flagellar motility gene promoters, allowing transcription of genes associated with flagellar motility at low temperatures (Kamp and Higgins, 2011). In summary, we propose that TrxA as a vital cellular reductase is essential for maintaining a highly reducing environment in the bacterial cytosol, which provides a favorable condition for protein folding and activation, especially for the site-specific DNA-binding transcription regulators whose activation needs merization, and thus is involved in a range of biological functions.

In addition, TrxA expression might be regulated by the alternative sigma factor, SigH, through binding to the promoter region of thioredoxin system genes by EMSA assay. Actually, previous microarray assay has shown that *L. monocytogenes trxA* and *trxB* exhibited 1.5-fold higher transcript levels in the WT than in the ΔSigH strain (Chaturongakul et al., 2011). The role of SigH in oxidative stress was first demonstrated by Fernandes and co-workers using *M. smegmatis sigH* mutants. The group showed that SigH protects against oxidative stress by regulating the expression of thioredoxins (*trxB1* and *trxC*) and thioredoxin reductase (Fernandes et al., 1999). Under reducing conditions, RshA, an anti-sigma factor, binds SigH, and inhibits the transcription of Trx and TrxR. However, under oxidative stress conditions, SigH is released from the complex and binds the promoters of Trx and TrxR to activate their transcription (Lu and Holmgren, 2014a). Consequently, we speculate that no expression or constitutive overexpression of TrxA *in vivo* would disturb the equilibrium of the intracellular redox potential and thus bring harm to *L. monocytogenes* for its ability to establish infections.

Generally, stress-tolerant *Listeria* strains are more invasive *in vitro* (Conte et al., 2000) and more virulent *in vivo* (Gahan et al., 1996; Cheng et al., 2015). Obviously, the capacity to tolerate oxidative stress is related to the virulence potential of *L. monocytogenes* and other bacterial species (Degnan et al., 2000; Chen et al., 2011). We demonstrated that the oxidative resistance ability is closely linked to *L. monocytogenes* pathogenicity, providing molecular insights into why the TrxA mutant is attenuated and nonmotile. Overall, our data should aid in clarifying the redox modification mechanisms of the thioredoxin system utilized by *L. monocytogenes* to adapt to niche environments outside and inside the host with a view to pathogen infection control from the public health perspective.

## Materials and methods

### Bacterial strains, plasmids, primers, and culture conditions

*L. monocytogenes* EGD-e was used as the wild-type strain. *E. coli* DH5α was employed for cloning experiments and as the host strain for plasmids pET30a(+) (Merck, Darmstadt, Germany), pIMK2 and pKSV7. *E. coli* Rosetta (DE3) was used for prokaryotic protein expression. *Listeria* strains were cultured in brain heart infusion (BHI) medium (Oxoid, Hampshire, England). *E. coli* strains were grown at 37°C in Luria-Bertani broth (LB) (Oxoid). Stock solutions of ampicillin (50 mg/ml), erythromycin (50 mg/ml), kanamycin (50 mg/ml), or chloramphenicol (50 mg/ml) were added to media when necessary. All chemicals were obtained from Sangon Biotech (Shanghai, China), Merck or Sigma-Aldrich (St. Louis, USA) and were of the highest available purity. All primers used in this study are listed in Table S3.

### Bioinformatics analysis

The amino acid sequences of putative thioredoxin proteins from *L. monocytogenes* EGD-e and its homologs in other microbial species were obtained from the National Centre for Biotechnology Information database (NCBI). Trx amino acid sequences were aligned with MUSCLE using Geneious software (Edgar, 2004). The phylogenetic tree was constructed with the Neighbor-joining (NJ) method using 100 bootstrap replicates.

### Prokaryotic expression and purification of recombinant proteins

The recombinant proteins used in this study were expressed as an N-terminal His tag fusion using pET30a(+) as the expression vector and Rosetta (DE3) as the expression host. The full-length ORF of the gene of interest from the EGD-e genome was amplified with the primer pair, inserted into the pET30a(+) vector, and finally transformed into Rosetta competent cells. *E. coli* cells harboring recombinant plasmids were grown in 500 mL LB supplemented with 50 μg/mL kanamycin at 37°C until cultures reached 0.8–1.0 at OD_600nm_. Isopropyl β-D-1-thiogalactopyranoside (IPTG) was added to a final concentration of 0.2 mM to induce expression of recombinant proteins for an additional 3 h under optimized conditions. His-tagged fusion proteins were purified using nickel-chelated affinity column chromatography.

### Preparation of polyclonal antibodies against recombinant proteins

Rabbits were initially immunized via subcutaneous injection of 500 μg protein with an equal volume of Freund's complete adjuvant (Sigma). After 2 weeks, rabbits were boosted by subcutaneous injection of 250 μg protein each in incomplete Freund's adjuvant (Sigma) three times at 10 day intervals. Rabbits were bled ~10 days after the last injection.

### Site-directed mutagenesis

To identify the predicted active sites of TrxA, single (C28A and C31A) and a double mutant (C28AC31A) were generated using the original vector template, pET30a-TrxA, and the QuikChange Site-Directed Mutagenesis kit (Agilent Technologies, Palo Alto, USA) with the primer pairs described in Table S3. Template DNA was removed via digestion with DpnI (Toyobo Co., Osaka, Japan) for 2 h at 37°C. All mutant constructs were sequenced to ensure that only the desired single mutations had been incorporated correctly. The corresponding mutant proteins were designated TrxA_C28A, TrxA_C31A, and TrxA_C28AC31A, and expressed and purified as described above.

### *In vitro* oxidoreductase activity assays

The ability of TrxA, DsbA, and DsbC/G to catalyze reduction of human insulin (Sigma) in the presence of DTT was measured as described previously by Holmgren (1979). Briefly, reaction mixtures were prepared using 0.1 M potassium phosphate buffer (pH 7.0), 150 μM insulin, 2 mM EDTA, and 1.0 μM purified proteins in a final volume of 200 μL. Reactions were initiated by adding DTT to a final concentration of 1 mM, and monitored as the increase in absorbance at 650 nm every 3 min (100 min in total) at 25°C using the Micro-plate reader Synergy H1 (BioTek Solutions, Inc., Santa Barbara, USA). The calibration curve for quantitative analysis of reduced insulin was determined by incubating 0.5 μM purified TrxA in PBS containing 1 mM DTT with various concentrations of insulin (0, 40, 80, 120, 160, and 200 μM).

### Construction of gene deletion mutants

Construction of *L. monocytogenes* gene deletion mutants were performed as described previously (Camilli et al., 1993). The temperature-sensitive pKSV7 shuttle vector was used for generating mutations in the *L. monocytogenes* strain EGD-e. A homologous recombination strategy with the splicing by overlap extension (SOE) PCR procedure was used for in-frame deletion to construct gene deletion mutants (Camilli et al., 1993; Monk et al., 2008). Specifically, DNA fragments containing homologous arms upstream and downstream of the gene of interest were obtained via amplification of EGD-e genomic DNA using SOE primer pairs. The obtained fragment was then cloned into the temperature-sensitive shuttle vector pKSV7 and transformed into *E. coli* DH5α. After confirmation by sequencing, the recombinant vector containing the target gene deletion cassette was then electroporated into the competent *L. monocytogenes* EGD-e cells. Transformants were selected on BHI agar plates containing chloramphenicol (10 μg/ml). A single transformant was serially passaged for ~30 generations at a non-permissive temperature (40°C) in BHI medium containing 10 μg/ml chloramphenicol to promote chromosomal integration. A single colony with chromosomal integration was successively passaged in BHI medium without antibiotic at a permissive temperature (30°C) for ~50 generations to enable plasmid excision and curing. The recombinants were identified as chloramphenicol-sensitive colonies, and the mutagenesis was further confirmed by PCR and DNA sequencing.

### Complementation of gene deletion mutants

To complement the *L. monocytogenes* Δ*trxA* strain, we constructed two complement strains using the integrative plasmid, pIMK2, which harbors a constitutive *Listeria* promoter P_*help*_ (Monk et al., 2008). For the first complement strain, we amplified the complete open reading frame (ORF) of *trxA* from EGD-e genomic DNA using the primer pair CΔ*trxA*-a/CΔ*trxA*-b and inserted the fragment downstream of P_*help*_ after digestion with the appropriate restriction enzymes. For the other complement strain, the complete ORF of *trxA* along with its promoter region was amplified using the primer pair CΔ*trxA*-c/CΔ*trxA*-d and cloned into pIMK2 following digestion with the appropriate restriction enzymes to remove the P_*help*_ region. The resulting plasmids were electroporated into the *L. monocytogenes* Δ*trxA* strain. Regenerated cells were plated on BHI agar containing kanamycin (50 μg/ml). The two complement strains were designated CΔ*trxA*_P_*help*_ and CΔ*trxA*_P_*trxA*_, respectively.

### Growth analysis of *L. monocytogenes* under oxidative conditions

*L. monocytogenes* wild-type strain EGD-e, mutant Δ*trxA* and two complement strains, CΔ*trxA*_P_*help*_ and CΔ*trxA*_P_*trxA*_, were grown overnight at 37°C in BHI broth with shaking. Cultures were collected by centrifugation at 5,000 × g at 4°C, washed once in PBS (10 mM, pH 7.4) and initial OD_600 nm_ adjusted to 1.0. Bacteria were diluted (1:100) in fresh BHI broth or BHI containing 5 mM paraquat, 4 mM diamide, or 10 mM H_2_O_2_, and incubated at 37°C for 12 h. Kinetic growth at OD_600 nm_ was measured at 1 h intervals.

### Changes in TrxA and TrxB expression levels under oxidative conditions

Bacteria were grown in BHI broth to the stationary growth phase and lysates prepared as described earlier (Ryan et al., 2009). Specifically, stationary bacteria were collected at the indicated times after treatment with 5 mM paraquat, 4 mM diamide, or 10 mM H_2_O_2_. Bacterial pellets were re-suspended in 1 mL of extraction solution (2% Triton X-100, 1% SDS, 100 mM NaCl, 10 mM Tris-HCl, 1 mM EDTA, pH 8.0). One gram of glass beads (G8772, Sigma) was added and samples lysed using the homogenizer Precellys 24 (Bertin, Provence, France) at 6,000 rpm for 30 s with intermittent cooling for 30 s (2 cycles in total), followed by centrifugation at 12,000 g for 15 min. Supernatants were retained as cell-free extracts. Proteins were separated via 12% SDS-PAGE and blotted onto 0.22 μm PVDF membrane (Merck). Membranes were blocked for 1 h with 5% skimmed milk, and incubated for 1 h with polyclonal antisera against recombinant TrxA or TrxB (prepared in this study) in 0.5% skimmed milk. Next, membranes probed with anti-TrxA or anti-TrxB were developed using HRP-conjugated goat anti-rabbit IgG (Santa Cruz, California, USA) as the secondary antibody. Chemiluminescence was detected *via* an UVP EC3 imaging system (UVP Inc., Upland, USA).

### Motility assay (swimming ability) and transmission electron microscopy (TEM)

Motility assay was performed essentially on soft LB agar (0.25%) as described (Paxman et al., 2009). *L. monocytogenes* were grown overnight in BHI and the cultures adjusted at OD_600 nm_ to 0.40 (about 2 × 10^8^ CFU/ml). Bacterial samples (5 μL) were dropped onto soft TSA agar and incubated at 30° or 37°C for 48 h to allow growth. Motility was assessed by examining migration of bacteria through agar from the center toward the periphery of the colony. TEM experiments were performed as described previously (Dingle et al., 2011) in the Institute of Agrobiology and Environmental Sciences of Zhejiang University. Briefly, *L. monocytogenes* colonies grown overnight at 30°C from BHI agar plates were suspended in 50 μL monoethanolamine buffer (pH 10.0), and 10 μL of the suspension applied to carbon-coated copper grids and allowed to stand for 2 min at room temperature. Excess liquid was subsequently removed using filter paper, and bacteria stained with 10 μL of 0.5% phosphotungstic acid (pH 7.0) placed on the grids for 10 s at room temperature. Excess stain was gently wicked away using filter paper, and the dried grids examined under a Hitachi H-7650 transmission electron microscope (Hitachi High-Technologies Corporation, Tokyo, Japan).

### Determination of protein-ligand interactions via isothermal titration calorimetry (ITC)

The protein-protein interactions between TrxA (or its active site mutants), MogR, and GmaR were measured using isothermal titration calorimetry (ITC) with a VP-isothermal titration calorimeter from Microcal, Inc. (Northampton, USA). Calorimetric titrations of TrxA (80–200 μM in the syringe) and PrfA, MogR, or GmaR (8–20 μM in the cuvette) were carried out at 25°C in 25 mM Tris-HCl, pH 7.5, 100 mM NaCl. TrxA or its active site mutants (C28A, C31A, and C28AC31A) was titrated into PrfA, MogR, or GmaR in 15 μL injections with a spacing of 300 s between injections. Calorimetric data were analyzed by integrating heat effects normalized to the amount of injected protein and curve-fitting based on a 1:1 binding model using the Origin software package (Microcal). The dissociation constant was derived from data using standard procedures. ITC was carried out in the Life Sciences Institute of Zhejiang University.

### *In vitro* redox status determination of MogR with TrxA

Reactions with recombinant TrxA were carried out in 50 mM Tris-HCl buffer, pH 7.5 with the increasing amount (0–2 μg) of reduced TrxA and 2 μg MogR with or without 0.1 mM DTT as an electron donor. After 1 h incubation, an equal amount of SDS–PAGE sample buffer was added to quench the reactions. Reactions were terminated with 10% (v/v) trichloroacetic acid (TCA) and then separated on 12% polyacrylamide gels, and stained with Coomassie blue to visualize the redox state of MogR.

### Cadmium and copper sensitivity assays

Cadmium and copper sensitivity assays are usually performed to test oxidase and isomerase activities, respectively (Ren et al., 2014). *L. monocytogenes* wild-type strain EGD-e, isogenic *lmo1059* and *lmo0964* deletion mutants and the corresponding complementation strains were grown overnight in BHI broth. Bacteria were diluted to OD_600nm_ of 1.0 (~10^9^ cfu ml^−1^) in PBS. Bacteria were serially diluted 10-fold, and 10 μl of each dilution spotted onto BHI agar plates containing 1 mM cadmium chloride (Sangon) or 1 mM copper(II) chloride (Sangon). Following incubation at 37°C for 48–72 h, colony growth on each plate was assessed and imaged. All spot titer assays were performed in duplicate to verify the results.

### Disulfide interchange reactions

Disulfide interchange reactions were used to investigate the ability of TrxA, DsbA, and DsbC/G to catalyze the reduction of oxidized glutathione (GSSG) by DTT. The DTT/glutathione equilibrium lies far on the side of oxidized DTT, and DTT_ox_ formation can be followed spectrophotometrically. GSSG (5 mM) was reduced by DTT (5 mM) at 25°C in 100 mM formic acid/NaOH (pH 4.0), 1 mM EDTA. Reactions were monitored based on the increase in absorbance at 287 nm.

### Comparison of transcriptome profiles of wild-type and the isogenic *trxA* mutant upon exposure to oxidative stress

#### RNA preparation

Bacterial overnight cultures of wild-type EGD-e and its isogenic *trxA* mutant were diluted (1:100) in fresh BHI broth and grown to the early stationary phase. Pellets were centrifuged at 5,000 g for 10 min, re-suspended in equal volumes of fresh BHI containing 2 mM diamide, and incubated statically at 37°C for additional 1 h. Total RNA was extracted using the Column Bacterial total RNA Purification Kit (Sangon), according to the manufacturer's instructions, and genomic DNA removed using DNase I (TaKara, Japan). RNA purity was assessed using the NanoDrop (Thermo Fisher Scientific, Lafayette, USA) and RNA integrity determined via electrophoresis.

#### Transcriptome sequencing

RNA samples were sent to the commercial sequencing company, Majorbio, for library construction and transcriptome sequencing, based on the Illumina Hiseq2500 sequencing platform. The paired-end raw reads were trimmed and quality controlled with SeqPrep (https://github.com/jstjohn/SeqPrep) and Sickle (https://github.com/najoshi/sickle) using default parameters. Clean reads were separately aligned to the reference genome with the orientation mode using Bowtie 2 software (Langmead and Salzberg, 2012). Output data generated from sequencing were stored in the standard FASTQ format for use as inputs for subsequent analyses.

#### Differential expression analysis and functional annotation

RSEM (RNA-Seq by Expectation-Maximization; Li and Dewey, 2011) was used to quantify gene and isoform abundance. EdgeR (Empirical analysis of digital gene expression data in R) (Robinson et al., 2010) was utilized for differential expression analysis. The TMM (trimmed mean of *M*-values) method was selected to compute normalization factors and differentially expressed genes (DEGs) between two samples selected using the following criteria: (I) logarithmic of fold change >1.0 and (II) FDR (false discovery rate) < 0.05. To determine the functions of the differentially expressed genes, the unigenes were aligned by BLASTx against the NCBI non-redundant (nr), Swiss-Prot, Kyoto Encyclopedia of Genes and Genome (KEGG), and Cluster of Orthologous Groups (COG) protein databases. GO functional enrichment analyses were carried out using Goatools and KOBAS (Xie et al., 2011). DEGs were significantly enriched in GO terms and metabolic pathways at Bonferroni-corrected *P*-values of < 0.05.

### qRT-PCR validation of transcriptome results

To validate of the reliability of transcriptome sequencing data, real-time quantification of random mRNAs was performed using qRT-PCR with the same total cellular RNAs used for transcriptome experiments. We randomly selected 28 genes for qRT-PCR validation, i.e., 16 representative downregulated genes and 12 representative upregulated genes in the Δ*trxA* mutant. Specific primers for these genes were designed and purchased from Sangon Biotech. cDNA was synthesized with reverse transcriptase (Toyobo). Quantitative PCR was performed in 20 μL reaction mixtures containing SYBR quantitative PCR mix (Toyobo) to measure the transcriptional levels of genes of interest using the Mx3000P PCR detection system (Agilent) with specific primer pairs. Real-time quantitative PCR was performed in a 20 μL reaction volume containing 200 ng cDNA, 10 μL SYBR quantitative PCR mix and 1 μL gene-specific primers (200 nM). The housekeeping gene, *rpoB*, was used as an internal control for normalization in each sample. Triplicate assays were performed for each gene.

### Changes in expression levels of LLO in the absence of *trxA*

To further establish, whether TrxA is involved in bacterial virulence, western blot was employed to analyze the changes in expression of the major virulence-associated factor, LLO. Bacterial overnight cultures of *L. monocytogenes* wild-type EGD-e, mutant Δ*trxA* and two complement strains, CΔ*trxA*_P_*help*_ and CΔ*trxA*_P_*trxA*_, were diluted into 100 mL fresh BHI broth and grown to the stationary phase. For isolation of secreted proteins, the fractionation procedure was performed as described before (Lenz and Portnoy, 2002), with minor modifications. Briefly, bacterial cells were pelleted via centrifugation at 13,000 g for 20 min at 4°C, and the resulting culture supernatant collected and filtered through a 0.22 μm polyethersulfone membrane filter (Merck). Trichloroacetic acid (TCA) was added to the supernatant to obtain a final concentration of 10%. Proteins were TCA-precipitated on ice overnight and washed with ice-cold acetone. Washed precipitates of supernatant proteins were re-suspended in SDS-PAGE sample buffer (5% SDS, 10% glycerol, and 50 mM Tris-HCl, pH 6.8). Samples were boiled for 5 min and stored at -20°C before electrophoresis. Total cell protein isolation was performed as described above. Protein samples were separated using 12% SDS-PAGE and immunoblotted with α-LLO or α-GAPDH antisera (prepared in this study). GAPDH was employed as an internal control.

### LLO-mediated hemolytic activity measurement

Measurement of LLO-associated hemolytic activity was done as described previously (Xayarath et al., 2015). *L. monocytogenes* wild-type and mutant strains were grown for 16 h with shaking in BHI broth at 37°C. All cultures were adjusted to OD_600nm_ of 1.0 before supernatants were collected. Hemolytic activity was measured based on lysis of sheep red blood cells (SRBCs) using supernatants from cultures. The culture supernatant (50 μL) was diluted with an equal volume of PBS containing 6 mM cysteine (pH 5.6) and equilibrated to 37°C for 10 min. Next, 50 μL PBS-washed intact SRBCs were added to each sample and incubated at 37°C for 60 min. Samples were centrifuged and supernatants analyzed for hemoglobin absorption at 550 nm. PBS was employed as a negative control. The values corresponding to the reciprocal of the dilution of culture supernatant required to lyse 50% of HRBCs were used to compare the hemolytic activities in the different supernatants.

### Adhesion, invasion, and survival in Caco-2 cells

Bacterial survival in human intestinal epithelial Caco-2 cells was assessed as described previously (Reddy et al., 2016). Stationary *L. monocytogenes* wild-type strain EGD-e, mutant Δ*trxA* and two complement strains, CΔ*trxA*_P_*help*_ and CΔ*trxA*_P_*trxA*_, at 37°C in BHI were washed and re-suspended in PBS (10 mM, pH 7.4). Caco-2 cells cultured to 80% confluence in Dulbecco's modified eagle medium (DMEM) containing 10% fetal calf serum were infected with the above strains for 60 min at a multiplicity of infection (MOI) of 10:1. For adhesion, cells were lysed after being washed three times with PBS. For estimation of invasion, cells were washed with PBS after 1 h infection and incubated for an additional hour in DMEM containing gentamicin at 200 μg/mL. Caco-2 cells were lysed by adding 1 mL of ice-cold sterile distilled water. Lysates were 10-fold diluted for enumeration of viable bacteria on BHI agar plates that were considered the 0 h numbers invading cells. For intracellular proliferation, cells were further incubated for 6 or 12 h in 5% CO_2_ at 37°C. Viable bacteria were enumerated as described above. Adhesion was expressed as the ratio of recovered colonies to colonies inoculated, while invasion was calculated as the ratio of colonies recovered after gentamicin treatment to colonies inoculated.

### Actin-tail formation in Caco-2 cells

Cells were infected at MOI of 10:1 at 37°C with 5% CO_2_ for 1 h. Extracellular bacteria were then killed with 200 μg/mL gentamicin for 1 h and incubated for an additional 6 h. Cells were washed gently with PBS (10 mM, pH 7.4), fixed with 4% paraformaldehyde and then permeabilized with 0.5% Triton X-100. The bacterial cells were stained with polyclonal antibodies to *L. monocytogenes* for 1 h at 37°C, washed twice with PBS and probed with Alexa Fluor 488-conjugated donkey anti-rabbit antibody (Santa Cruz) for 1 h at 37°C. F-actin was then stained with phalloidin-Alexa Fluor 568 (Thermo Fisher Scientific). DAPI (4′,6-diamidino-2-phenylindole; Thermo Fisher Scientific) was used to stain the nuclei. Actin tails recruited by the bacteria were visualized under a ZEISS LSM510 confocal microscope (Zeiss Germany, Oberkochen, Germany).

### Virulence in the mouse model

The *L. monocytogenes* wild-type strain EGD-e, mutant Δ*trxA* and two complement strains, CΔ*trxA*_P_*help*_ and CΔ*trxA*_P_*trxA*_, were tested for recovery in liver and spleen sections of ICR mice (female, 18–22 g, purchased from Zhejiang Academy of Medical Sciences, Hangzhou, China). ICR mice (eight per group) were injected intraperitoneally with ~10^6^ CFU of each strain. At 24 and 48 h post-infection, mice were sacrificed, and livers and spleens removed and individually homogenized in PBS (10 mM, pH 7.4). Surviving *Listeria* cells were enumerated by plating serial dilutions of homogenates on BHI agar plates. Results were expressed as means ± SD of the recovery rate per organ for each group.

### Electrophoretic mobility shift assay (EMSA)

To further demonstrate, whether the *L. monocytogenes* Trx system is regulated by SigH, we determined the binding abilities between SigH and promoter regions of *trxA* and *trxB in vitro* using the electrophoretic mobility shift assay (EMSA). DNA fragments of the promoter regions of *trxA* and *trxB* were generated by PCR with the specific primer pairs, and purified with the PCR Purification Kit (Sangon) according to the manufacturer's instructions. Next, 200 ng DNA was incubated with varying concentrations of purified recombinant SigH and incubated in binding buffer (50 mM Tris-HCl, pH 8.0, 250 mM NaCl, 5.0 mM MgCl_2_, 2.5 mM DTT, 2.5 mM EDTA, and 20% glycerol) for 30 min at room temperature. Protein-DNA complexes were separated electrophoretically on a native 5% polyacrylamide gel at 80 V with 0.5 × Tris-acetate-EDTA (TAE) buffer and visualized using ethidium bromide staining.

### Statistical analysis

All experiments were repeated at least three times. Data were analyzed using the two-tailed homoscedastic Student's *t*-test. Differences with *P* < 0.05 were considered statistically significant.

### Ethics statements

All animal care and use protocols were performed in accordance with the Regulations for the Administration of Affairs Concerning Experimental Animals approved by the State Council of People's Republic of China. The protocol was approved by the Institutional Animal Care and Use Committee of Zhejiang A&F University (Permit Number: ZJAFU/IACUC_2011-10-25-02).

## Author contributions

CC, WF, and HS conceived the study. CC, JS, XH, ZD, HW, LJ, CS, and TM carried out experiments. CC, YY, ZC, HH, and XW analyzed data. CC, WF, NF, and HS drafted the manuscript and all the authors contributed to preparing the final version of the manuscript. All authors read and approved the final manuscript.

### Conflict of interest statement

The authors declare that the research was conducted in the absence of any commercial or financial relationships that could be construed as a potential conflict of interest.

## Supplementary material

Figure S1 The thioredoxin system is regulated by SigH. SigH bound to *trxA* and *trxB* promoters, as determined using the electrophoretic mobility shift assay. P_*trxA*_ and P_*trxB*_ fragments were obtained by PCR with the primers specified in Table S3, and incubated with recombinant SigH for 30 min at room temperature. Gel retardation of DNA-protein complexes was monitored after ethidium bromide staining. The promoter DNA fragment of *rpoB* (a house-keeping gene in *L. monocytogenes*, which is annotated as DNA-directed RNA polymerase subunit beta) was considered as a negative control.

Figure S2 Lmo0964 (DsbA) is responsible for bacterial motility. The motility assay was performed by growing *L. monocytogenes* WT EGD-e, Δ*lmo1059*, Δ*lmo0964* and the complement strains, CΔ*lmo1059* and CΔ*lmo0964*, on soft agar (0.25%) at 30°C.

Table S1 Genes identified as significantly downregulated in the mutant strain of *L. monocytogenes*, Δ*trxA*, via transcriptome analysis.

Table S2 Genes identified as significantly upregulated in the mutant strain of *L. monocytogenes*, Δ*trxA*, via transcriptome analysis.

Table S3 PCR primers used in this study. The nucleotides introduced to create restriction enzyme sites are underlined. All primers were synthesized by Sangon Biotech, Inc., Shanghai, China.
