# Supplementary figures and images for: Thioredoxin A Is Essential for Motility and Contributes to Host Infection of Listeria monocytogenes via Redox Interactions
Source: Front Cell Infect Microbiol. 2017 Jun 28;7:287. doi: 10.3389/fcimb.2017.00287 (PMC5487381; doi:10.3389/fcimb.2017.00287)

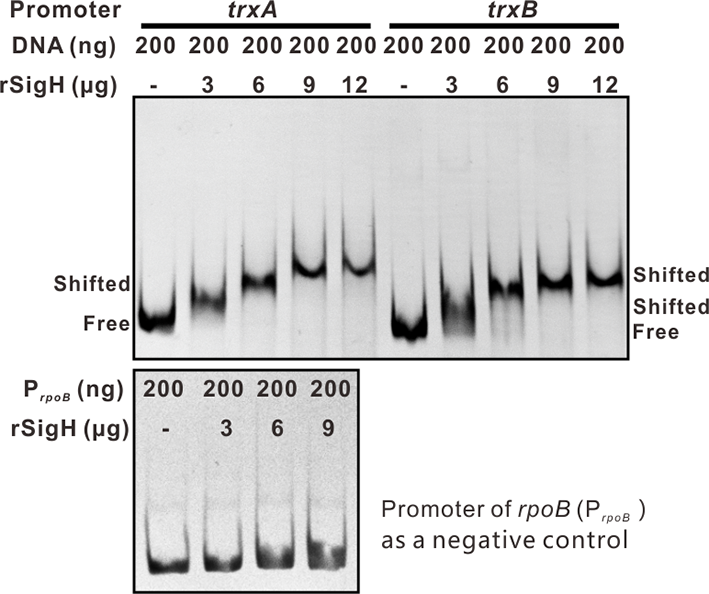

Supplement: Figure S1 — The thioredoxin system is regulated by SigH. SigH bound to trxA and trxB promoters, as determined using the electrophoretic mobility shift assay. PtrxA and PtrxB fragments were obtained by PCR with the primers specified in Table S3, and incubated with recombinant SigH for 30 min at room temperature. Gel retardation of DNA-protein complexes was monitored after ethidium bromide staining. The promoter DNA fragment of rpoB (a house-keeping gene in L. monocytogenes, which is annotated as DNA-directed RNA polymerase subunit beta) was considered as a negative control. [file Image1.TIF]

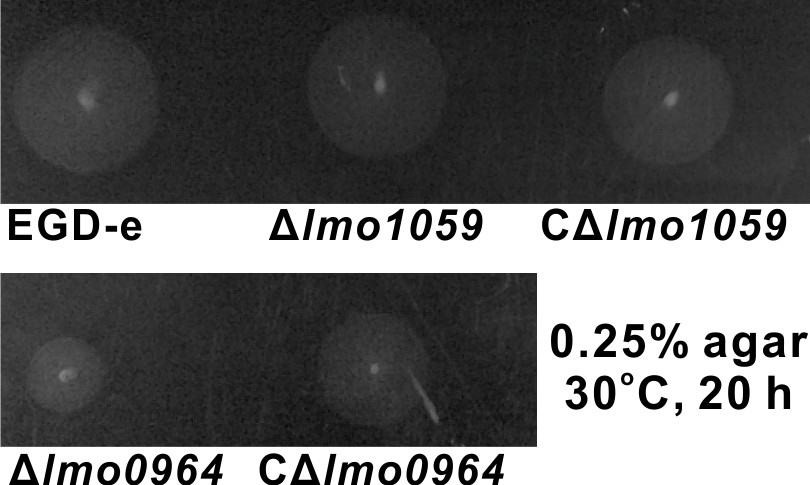

Supplement: Figure S2 — Lmo0964 (DsbA) is responsible for bacterial motility. The motility assay was performed by growing L. monocytogenes WT EGD-e, Δlmo1059, Δlmo0964 and the complement strains, CΔlmo1059 and CΔlmo0964, on soft agar (0.25%) at 30°C. [file Image2.TIF]
