# Supplementary material for: Thioredoxin A Is Essential for Motility and Contributes to Host Infection of Listeria monocytogenes via Redox Interactions
Source: Front Cell Infect Microbiol. 2017 Jun 28;7:287. doi: 10.3389/fcimb.2017.00287 (PMC5487381; doi:10.3389/fcimb.2017.00287)
Supplement: Table S1 — Genes identified as significantly downregulated in the mutant strain of L. monocytogenes, ΔtrxA, via transcriptome analysis. [file Table1.PDF]

Table S1. Genes identified as significantly downregulated in the mutant strain of *L. monocytogenes*, Δ*trxA*, via transcriptome analysis.

Locus tag	Gene name	Protein	Fold change	Significance
lmo1233	*trxA*	thioredoxin	1424.54	yes
lmo1249	*lmo1249*	hypothetical protein	77.10	yes
lmo1250	*lmo1250*	quinolone MFS transporter	52.28	yes
lmo0115	*lmo0115*	hypothetical protein	20.75	yes
lmo2850	*iolF*	Minor myo-inositol transporter iolF	18.91	yes
lmo0118	*lmo0118*	hypothetical protein	18.61	yes
lmo0116	*lmo0116*	hypothetical protein	17.82	yes
lmo1251	*lmo1251*	Crp/Fnr family transcriptional regulator	16.90	yes
lmo0641	*zntA*	ATPase	16.85	yes
lmo0120	*lmo0120*	hypothetical protein	14.77	yes
lmo0121	*lmo0121*	membrane protein	14.28	yes
lmo0128	*lmo0128*	hypothetical protein	12.22	yes
lmo0129	*lmo0129*	N-acetylmuramoyl-L-alanine amidase	11.53	yes
lmo0117	*lmo0117*	hypothetical protein	11.48	yes
lmo0122	*lmo0122*	hypothetical protein	10.80	yes
lmo0123	*lmo0123*	hypothetical protein	10.57	yes
lmo0127	*lmo0127*	hypothetical protein	10.33	yes
lmo0125	*lmo0125*	hypothetical protein	10.29	yes
lmo2841	*lmo2841*	sucrose phosphorylase	10.21	yes
lmo0126	*lmo0126*	hypothetical protein	10.17	yes
lmo0203	*mpl*	peptidase M4	8.55	yes
lmo0119	*lmo0119*	hypothetical protein	7.76	yes
lmo0124	*lmo0124*	hypothetical protein	6.76	yes
lmo2306	*lmo2306*	hypothetical protein	6.46	yes

lmo2849	*rhaB*	rhamnulokinase	6.11	yes
lmo2317	*lmo2317*	hypothetical protein	6.11	yes
lmo2785	*kat*	catalase	6.04	yes
lmo0280	*nrdG*	anaerobic ribonucleoside-triphosphate reductase activating protein	5.80	yes
lmo2836	*lmo2836*	alcohol dehydrogenase	5.20	yes
lmo0279	*nrdD*	ribonucleoside triphosphate reductase	5.12	yes
lmo0835	*lmo0835*	peptidoglycan-binding protein	4.74	yes
lmo1654	*lmo1654*	hypothetical protein	4.66	yes
lmo0205	*plcB*	phospholipase C	4.58	yes
lmo2151	*lmo2151*	hypothetical protein	4.55	yes
lmo0348	*dhaK*	dihydroxyacetone kinase subunit K	4.50	yes
lmo0032	*lmo0032*	ROK family transcriptional regulator	4.38	yes
lmo0204	*actA*	actin assembly-inducing protein	4.33	yes
lmo2323	*lmo2323*	hypothetical protein	4.29	yes
lmo2172	*pct*	CoA transferase	4.25	yes
lmo0057	*lmo0057*	hypothetical protein	4.08	yes
lmo0315	*tenA*	Listeria thiamin biosynthesis protein	4.07	yes
lmo1917	*pflA*	formate acetyltransferase	4.05	yes
lmo2319	*lmo2319*	hypothetical protein	4.00	yes
lmo0380	*lmo0380*	hypothetical protein	3.98	yes
lmo0838	*uhpT*	antiporter	3.88	yes
lmo2816	*lmo2816*	MFS transporter	3.87	yes
lmo0201	*plcA*	1-phosphatidylinositol phosphodiesterase	3.84	yes
lmo2446	*lmo2446*	glycosyl hydrolase family 31	3.81	yes
lmo0026	*cutC*	copper homeostasis protein CutC	3.76	yes
lmo2708	*celB*	PTS cellobiose transporter subunit IIC	3.63	yes
lmo2328	*lmo2328*	XRE family transcriptional regulator	3.60	yes
lmo1969	*eda*	2-keto-3-deoxygluconate-6-phosphate aldolase	3.52	yes

lmo2171	*oxlT*	MFS transporter	3.45	yes
lmo2135	*lmo2135*	Listeria PTS fructose transporter subunit IIC	3.44	yes
lmo0383	*lmo0383*	methylmalonate-semialdehyde dehydrogenase	3.37	yes
lmo0025	*lmo0025*	Listeria phosphoheptose isomerase	3.29	yes
lmo0903	*lmo0903*	osmotically inducible protein C	3.21	yes
lmo2362	*gadT2*	glutamate:gamma-aminobutyrate antiporter	3.17	yes
lmo1191	*cbiA*	cobyrinic acid a,c-diamide synthase	3.17	yes
lmo1971	*ulaA*	PTS ascorbate transporter subunit IIC	3.16	yes
lmo0943	*dps*	general stress protein	3.13	yes
lmo2288	*lmo2288*	Listeria phage A118	3.13	yes
lmo2149	*lmo2149*	nucleotide pyrophosphohydrolase	3.12	yes
lmo2651	*lmo2651*	PTS sugar transporter subunit IIA	3.11	yes
lmo1192	*cobD*	cobalamin biosynthesis protein	2.97	yes
lmo2324	*lmo2324*	antirepressor	2.97	yes
lmo2646	*lmo2646*	hypothetical protein	2.96	yes
lmo0300	*lmo0300*	6-phospho-beta-galactosidase	2.94	yes
lmo2848	*rhaA*	rhamnose isomerase	2.93	yes
lmo0060	*lmo0060*	membrane protein	2.93	yes
lmo2688	*lmo2688*	cell division protein FtsW	2.92	yes
lmo2847	*rhaD*	rhamnulose-1-phosphate aldolase	2.92	yes
lmo0659	*lmo0659*	hypothetical protein	2.92	yes
lmo2327	*lmo2327*	hypothetical protein	2.86	yes
lmo0105	*E3.2.1.14*	Listeria chitinase B	2.86	yes
lmo2835	*lmo2835*	hypothetical protein	2.83	yes
lmo0033	*lmo0033*	Listeria endoglucanase	2.82	yes
lmo2681	*kdpB*	Potassium-transporting ATPase B	2.78	yes
lmo2131	*lmo2131*	cAMP-binding protein	2.75	yes
lmo0061	*lmo0061*	cell division protein FtsK	2.67	yes

lmo0310	*lmo0310*	hypothetical protein	2.66	yes
lmo1407	*pflA, pflC*	pyruvate formate lyase-activating protein	2.65	yes
lmo2676	lmo2676	type VI secretion protein ImpB	2.64	yes
lmo0431	lmo0431	Listeria acetyltransferase	2.63	yes
lmo2774	lmo2774	ABC transporter ATP-binding protein	2.63	yes
lmo0897	lmo0897	sulfate transporter	2.63	yes
lmo2682	*kdpA*	ATPase	2.63	yes
lmo1786	*InlC*	internalin C	2.62	yes
lmo0238	*cysE*	serine acetyltransferase	2.61	yes
lmo2301	*xtmA*	Listeria terminase	2.61	yes
lmo0159	*lmo0159*	peptidoglycan-binding protein	2.60	yes
lmo2152	*lmo2152*	thioredoxin	2.60	yes
lmo0326	*lmo0326*	transcriptional regulator	2.60	yes
lmo0353	*lmo0353*	GNAT family acetyltraansferase	2.60	yes
lmo0089	lmo0089	ATP synthase F1 subunit delta	2.58	yes
lmo2806	*lmo2806*	hypothetical protein	2.58	yes
lmo1406	*pflB*	pyruvate-formate lyase	2.54	yes
lmo2153	*lmo2153*	Listeria flavodoxin	2.53	yes
lmo2819	*lmo2819*	Listeria carboxypeptidase	2.52	yes
lmo2300	*xtmB*	Listeria terminase large subunit from bacteriophage A118	2.51	yes
lmo2363	*gadB*	glutamate decarboxylase	2.47	yes
lmo2213	*lmo2213*	antibiotic biosynthesis monooxygenase	2.47	yes
lmo2675	*lmo2675*	hypothetical protein	2.44	yes
lmo0239	*cysS*	cysteinyl-tRNA synthetase	2.43	yes
lmo0069	*lmo0069*	hypothetical protein	2.42	yes
lmo2154	*nrdF*	ribonucleotide-diphosphate reductase subunit beta	2.41	yes
lmo2781	*bglX*	Listeria beta-glucosidase	2.40	yes
lmo0912	*lmo0912*	MULTISPECIES: formate/nitrite transporter	2.39	yes

lmo1659	*lmo1659*	hypothetical protein	2.38	yes
lmo2828	*lmo2828*	hypothetical protein	2.37	yes
lmo0767	lmo0767	ABC transporter permease	2.36	yes
lmo1303	*lmo1303*	cell division suppressor protein YneA	2.35	yes
lmo2271	*lmo2271*	hypothetical protein	2.35	yes
lmo0087	*lmo0087*	hypothetical protein	2.34	yes
lmo2691	*lmo2691*	amidase	2.34	yes
lmo0223	*cysK*	cysteine synthase A	2.34	yes
lmo0446	lmo0446	Listeria penicillin acylase	2.33	yes
lmo2833	*ycjT*	glycosyl hydrolase family 65	2.31	yes
lmo2760	*lmo2760*	ABC transporter ATP-binding protein	2.31	yes
lmo0066	*lmo0066*	Listeria toxin	2.30	yes
lmo0152	lmo0152	peptide ABC transporter substrate-binding protein	2.27	yes
lmo0814	*fabK*	2-nitropropane dioxygenase	2.23	yes
lmo0839	*tetA*	MFS transporter [Listeria tetracycline resistance protein]	2.21	yes
lmo0251	*rplL*	50S ribosomal protein L7/L12	2.21	yes
lmo2689	lmo2689	magnesium-translocating P-type ATPase	2.20	yes
lmo0135	lmo0135	peptide ABC transporter substrate-binding protein	2.20	yes
lmo0241	*rlmB*	MULTISPECIES: RNA methyltransferase	2.20	yes
lmo0250	*rplJ*	50S ribosomal protein L10	2.19	yes
lmo2234	*lmo2234*	IolI protein, putative	2.19	yes
lmo0849	*amiE*	amidase	2.18	yes
lmo2409	*lmo2409*	hypothetical protein	2.16	yes
lmo0979	lmo0979	glycosyl transferase family 8	2.16	yes
lmo2669	*lmo2669*	membrane protein	2.16	yes
lmo0243	*sigH*	RNA polymerase sigma70 factor	2.15	yes
lmo0316	*thiM*	hydroxyethylthiazole kinase	2.15	yes
lmo0048	*agrB*	Listeria sensor histidine kinase AgrB	2.15	yes

lmo2337	lmo2337	DeoR family transcriptional regulator	2.14	yes
lmo2804	*lmo2804*	hypothetical protein	2.12	yes
lmo2818	lmo2818	quinolone resistance protein NorB	2.11	yes
lmo0179	lmo0179	sugar ABC transporter permease	2.11	yes
lmo0637	*lmo0637*	SAM-dependent methlyltransferase	2.11	yes
lmo0084	*lmo0084*	aldehyde oxidase	2.10	yes
lmo2586	*fdoG, fdfH*	formate dehydrogenase subunit alpha	2.10	yes
lmo0108	lmo0108	multidrug ABC transporter ATP-binding protein	2.10	yes
lmo1883	*lmo1883*	Listeria chitinase	2.08	yes
lmo0307	*lmo0307*	hypothetical protein	2.08	yes
lmo2236	*aroE*	shikimate 5-dehydrogenase	2.07	yes
lmo0242	*K06962*	hypothetical protein	2.05	yes
lmo1097	*lmo1097*	Listeria integrase	2.03	yes
lmo2856	*pmH*	50S ribosomal protein L34	2.03	yes
lmo0636	*lmo0636*	Rrf2 family transcriptional regulator	2.02	yes
lmo0646	lmo0646	glyoxalase	2.02	yes
lmo2336	*fruK*	Listeria fructose-1-phosphate kinase	2.02	yes
lmo2124	*ganP*	sugar ABC transporter permease	2.02	yes
lmo0611	*acpD*	Listeria azoreductase	2.02	yes
lmo2709	*lmo2709*	hypothetical protein	2.01	yes
lmo2772	*bglF*	PTS beta-glucoside transporter subunit IIA	2.01	yes
lmo0155	*znuB*	zinc ABC transporter permease	2.01	yes
lmo1274	*smf*	Listeria polypeptide deformylase	2.00	yes
lmo2187	*lmo2187*	hypothetical protein	2.00	yes
