# Supplementary material for: Thioredoxin A Is Essential for Motility and Contributes to Host Infection of Listeria monocytogenes via Redox Interactions
Source: Front Cell Infect Microbiol. 2017 Jun 28;7:287. doi: 10.3389/fcimb.2017.00287 (PMC5487381; doi:10.3389/fcimb.2017.00287)
Supplement: Table S2 — Genes identified as significantly upregulated in the mutant strain of L. monocytogenes, ΔtrxA, via transcriptome analysis. [file Table2.PDF]

**Table S2. Genes identified as significantly upregulated in the mutant strain of *L. monocytogenes*, Δ*trxA*, via transcriptome analysis.**

| Locus tag | Gene name | Protein | Fold change | Significance |
|---|---|---|---|---|
| lmo2185 | *lmo2185* | hypothetical protein | 54.28 | yes |
| lmo2186 | *lmo2186* | hypothetical protein | 24.61 | yes |
| lmo0541 | *lmo0541* | ABC transporter substrate-binding protein | 24.41 | yes |
| lmo0485 | *lmo0485* | nitroreductase | 19.13 | yes |
| lmo2184 | *lmo2184* | heme ABC transporter substrate-binding protein | 17.30 | yes |
| lmo2183 | *lmo2183* | ABC transporter permease | 16.86 | yes |
| lmo2180 | *lmo2180* | hypothetical protein | 16.76 | yes |
| lmo2182 | *lmo2182* | iron ABC transporter ATP-binding protein | 16.54 | yes |
| lmo2181 | *srtB* | sortase | 15.93 | yes |
| lmo0997 | *clpE* | Clp protease ClpX [Listeria Clp protease ClpX | 13.02 | yes |
| lmo1298 | *glnR* | HTH-type transcriptional regulator GlnR | 12.21 | yes |
| lmo0362 | *tatA* | preprotein translocase subunit TatA | 11.67 | yes |
| lmo1132 | *lmo1132* | ABC transporter ATP-binding protein | 11.53 | yes |
| lmo0367 | *efeB* | deferrochelatase | 9.56 | yes |
| lmo0366 | *efeO* | hypothetical protein | 7.96 | yes |
| lmo0484 | *isdG* | heme-degrading monooxygenase IsdG | 7.50 | yes |
| lmo0230 | *lmo0230* | excinuclease Uvr | 7.02 | yes |
| lmo1740 | *lmo1740* | amino acid ABC transporter permease | 6.75 | yes |
| lmo0231 | *lmo0231* | ATP:guanido phosphotransferase | 6.64 | yes |
| lmo2105 | *feoB* | ferrous iron transporter B | 6.64 | yes |
| lmo0229 | *ctsR* | MULTISPECIES: CtsR family transcriptional regulator | 6.62 | yes |
| lmo0361 | *tatC* | preprotein translocase subunit TatC | 6.45 | yes |
| lmo1960 | *fhuC* | iron-dicitrate ABC transporter ATP-binding protein | 6.37 | yes |
| lmo1517 | *glnB* | nitrogen regulatory PII | 6.18 | yes |

| | | | | |
|---|---|---|---|---|
| lmo0712 | *fliE* | flagellar hook-basal body protein FliE | 6.07 | yes |
| lmo1100 | *zntA* | cadmium transporter [Listeria cadmium resistance protein] | 6.00 | yes |
| lmo1138 | *clpP* | ATP-dependent Clp protease proteolytic subunit | 5.92 | yes |
| lmo1961 | *lmo1961* | ferredoxin--NADP reductase | 5.65 | yes |
| lmo1848 | *mtsB* | membrane protein [Listeria metal ABC transporter permease] | 5.45 | yes |
| lmo1131 | *lmo1131* | ABC transporter ATP-binding protein | 5.26 | yes |
| lmo0232 | *clpC* | class III stress response-related ATPase | 5.13 | yes |
| lmo0830 | *fbp* | fructose-1,6-bisphosphatase | 4.76 | yes |
| lmo1299 | *glnA* | glutamine synthetase | 4.63 | yes |
| lmo1993 | *pdp* | pyrimidine-nucleoside phosphorylase | 4.60 | yes |
| lmo1849 | *mtsC* | Listeria metal ABC transporter ATP-binding protein | 4.39 | yes |
| lmo2104 | *feoA* | Listeria ferrous iron transport protein A | 4.33 | yes |
| lmo1245 | *lmo1245* | hypothetical protein | 4.12 | yes |
| lmo2088 | *lmo2088* | transcriptional regulator, TetR family | 3.99 | yes |
| lmo1882 | *rpsN* | 30S ribosomal protein S14 | 3.88 | yes |
| lmo2210 | *lmo2210* | hypothetical protein | 3.77 | yes |
| lmo1959 | *lmo1959* | Listeria ferrichrome-binding protein | 3.76 | yes |
| lmo1516 | *nrgA* | ammonium transporter NrgA | 3.75 | yes |
| lmo1137 | *lmo1137* | hypothetical protein | 3.67 | yes |
| lmo1699 | *lmo1699* | chemotaxis protein | 3.58 | yes |
| lmo2468 | *clpP* | ATP-dependent Clp protease proteolytic subunit | 3.49 | yes |
| lmo0727 | *glmS* | glucosamine--fructose-6-phosphate aminotransferase | 3.47 | yes |
| lmo2437 | *lmo2437* | extradiol dioxygenase | 3.47 | yes |
| lmo1957 | *fhuG* | ferrichrome ABC transporter permease | 3.47 | yes |
| lmo2261 | *lmo2261* | MIP18 family protein yitW | 3.44 | yes |
| lmo0496 | *lmo0496* | hypothetical protein | 3.36 | yes |
| lmo1575 | *lmo1575* | oligoribonuclease | 3.31 | yes |
| lmo1958 | *fhuB* | ferrichrome ABC transporter permease | 3.27 | yes |

| | | | | |
|---|---|---|---|---|
| lmo0568 | *hisG* | ATP phosphoribosyltransferase | 3.27 | yes |
| lmo1634 | *adhE* | bifunctional acetaldehyde-CoA/alcohol dehydrogenase | 3.21 | yes |
| lmo0278 | *lmo0278* | sugar ABC transporter ATP-binding protein | 3.19 | yes |
| lmo2206 | *clpB* | protein disaggregation chaperone | 3.14 | yes |
| lmo1216 | *bax* | N-acetylmuramoyl-L-alanine amidase | 3.05 | yes |
| lmo0566 | *hisB* | imidazoleglycerol-phosphate dehydratase | 2.97 | yes |
| lmo2000 | *lmo2000* | Mannose permease IID component | 2.94 | yes |
| lmo0519 | *lmo0519* | lincomycin resistance protein LmrB | 2.87 | yes |
| lmo0567 | *hisD* | histidinol dehydrogenase | 2.82 | yes |
| lmo0785 | *lmo0785* | PTS sugar transporter subunit IIA | 2.76 | yes |
| lmo0977 | *lmo0977* | hypothetical protein | 2.74 | yes |
| lmo2522 | *lmo2522* | Listeria cell wall-binding protein | 2.71 | yes |
| lmo1387 | *proC* | Pyrroline-5-carboxylate reductase | 2.71 | yes |
| lmo2390 | *lmo2390* | ferredoxin--NADP reductase | 2.68 | yes |
| lmo0976 | *lmo0976* | Acetyltransferase | 2.67 | yes |
| lmo1576 | *lmo1576* | hypothetical protein | 2.67 | yes |
| lmo1739 | *lmo1739* | amino acid ABC transporter ATPase | 2.66 | yes |
| lmo1478 | *lmo1478* | MerR family transcriptional regulator | 2.63 | yes |
| lmo0398 | *lmo0398* | PTS fructose transporter subunit IIA | 2.62 | yes |
| lmo0622 | *lmo0622* | hypothetical protein | 2.60 | yes |
| lmo1439 | *sod* | superoxide dismutase | 2.59 | yes |
| lmo1881 | *lmo1881* | 5'-3' exonuclease | 2.57 | yes |
| lmo1870 | *lmo1870* | alkaline phosphatase | 2.51 | yes |
| lmo2460 | *cggR* | central glycolytic genes regulator | 2.50 | yes |
| lmo2087 | *lmo2087* | multidrug transporter MatE | 2.50 | yes |
| lmo0257 | *lmo0257* | hypothetical protein | 2.50 | yes |
| lmo0714 | *fliG* | flagellar motor switch protein FliG | 2.47 | yes |
| lmo1651 | *lmo1651* | multidrug ABC transporter ATP-binding protein | 2.45 | yes |

| | | | | |
|---|---|---|---|---|
| lmo0365 | *lmo0365* | hypothetical protein | 2.43 | yes |
| lmo0717 | *lmo0717* | transglycosylase | 2.42 | yes |
| lmo1847 | *mtsA* | manganese ABC transporter substrate-binding protein | 2.38 | yes |
| lmo2176 | *lmo2176* | TetR family transcriptional regulator | 2.37 | yes |
| lmo2411 | *sufB* | Fe-S cluster assembly protein SufB | 2.35 | yes |
| lmo1254 | *treC* | trehalose-6-phosphate hydrolase | 2.33 | yes |
| lmo0186 | *lmo0186* | hypothetical protein | 2.32 | yes |
| lmo1769 | *purQ* | phosphoribosylformylglycinamidine synthase | 2.32 | yes |
| lmo1243 | *phnB* | glyoxalase | 2.30 | yes |
| lmo0597 | *lmo0597* | cyclic nucleotide-binding protein | 2.29 | yes |
| lmo0560 | *gdhA* | glutamate dehydrogenase | 2.24 | yes |
| lmo0430 | *lmo0430* | LysR family transcriptional regulator | 2.24 | yes |
| lmo2412 | *nifU* | NifU-like protein | 2.24 | yes |
| lmo1771 | *lmo1771* | phosphoribosylformylglycinamidine synthase subunit PurS | 2.23 | yes |
| lmo1577 | *lmo1577* | metal-dependent hydrolase | 2.21 | yes |
| lmo1770 | *purL* | phosphoribosylformylglycinamidine synthase | 2.18 | yes |
| lmo0411 | *lmo0411* | phosphoenolpyruvate synthase | 2.18 | yes |
| lmo1859 | *msrB* | methionine sulfoxide reductase B | 2.17 | yes |
| lmo1738 | *lmo1738* | amino acid ABC transporter substrate-binding protein | 2.16 | yes |
| lmo1860 | *msrA* | methionine sulfoxide reductase A | 2.16 | yes |
| lmo0015 | *qoxC* | cytochrome O ubiquinol oxidase | 2.15 | yes |
| lmo1926 | *aroH* | chorismate mutase | 2.14 | yes |
| lmo1384 | *lmo1384* | hypothetical protein | 2.13 | yes |
| lmo2374 | *lysC* | aspartate kinase | 2.12 | yes |
| lmo1518 | *lmo1518* | hypothetical protein | 2.12 | yes |
| lmo2160 | *lmo2160* | xylose isomerase | 2.11 | yes |
| lmo1712 | *lmo1712* | Listeria multidrug resistance protein | 2.10 | yes |
| lmo1924 | *tyrA* | prephenate dehydrogenase | 2.07 | yes |

| | | | | |
|---|---|---|---|---|
| lmo1650 | *lmo1650* | membrane protein | 2.06 | yes |
| lmo2209 | *lmo2209* | GNAT family acetyltransferase | 2.05 | yes |
| lmo1983 | *ilvD* | dihydroxy-acid dehydratase | 2.05 | yes |
| lmo2742 | *lmo2742* | hypothetical protein | 2.03 | yes |
| lmo1006 | *patA* | aminotransferase A | 2.01 | yes |
| lmo2243 | *adaA* | AraC family transcriptional regulator | 2.01 | yes |
