# Supplementary material for: Thioredoxin A Is Essential for Motility and Contributes to Host Infection of Listeria monocytogenes via Redox Interactions
Source: Front Cell Infect Microbiol. 2017 Jun 28;7:287. doi: 10.3389/fcimb.2017.00287 (PMC5487381; doi:10.3389/fcimb.2017.00287)
Supplement: Table S3 — PCR primers used in this study. The nucleotides introduced to create restriction enzyme sites are underlined. All primers were synthesized by Sangon Biotech, Inc., Shanghai, China. [file Table3.PDF]

Table S3. PCR primers used in this study. The nucleotides introduced to create restriction enzyme sites are underlined. All primers were synthesized by Sangon Biotech, Inc., Shanghai, China.

Primer name	Primer sequence (5'-3')	Product (bp)	Description
Δ*trxA*-a	CGCGGATCCCGCGTCCTCTCACTTGGCC	523	Used for construction of *trxA* null mutant
Δ*trxA* -b	ATTTTAGCATAATTGATTTAATTCACTCCTTATGACTTTTGTACACAC		
Δ*trxA* -c	GAGTGAATTAAATCAATTATGCTAAAATGAAAGAAGCTTAGAACAAAC	519	
Δ*trxA* -d	AACTGCAGCGTTTTACTTCATTGGCAGCGTAG		
Δ*lmo1059*-a	TTTCCATGGATATTAGTCAAATTAAAGCAGA	548	Used for construction of *lmo1059* null mutant
Δ*lmo1059*-b	CATCATCAAGTAGCGAACGTAACTCTACTTCTGCTTTAATTTGACTAATATCCA		
Δ*lmo1059*-c	GAGTTACGTTCGCTACTTGATGATGAA	446	
Δ*lmo1059*-d	TTCAAGCTTTCATCTTCTTCAGAGAGCGCCGTCA		
Δ*lmo0964*-a	CGCGGATCCTAGAGTATGAAGTGTCTGATTATGACAAAGG	545	Used for construction of *lmo0964* null mutant
Δ*lmo0964*-b	GAAGAAGTAACTCAATTTGAAATAGTTCCCGTACTCCA		
Δ*lmo0964*-c	TATTTCAAATTGAGTTACTTCTTCTGCTTCTATGGCAGAT	522	
Δ*lmo0964*-d	GCACTGCAGACAAGCGATTATCTATATCGTTGCACT		
CΔ*trxA*-a	CATGCCATGGTAAAAGAAATTACAGATGCAACATTTG	327	Used for complementation of the *trxA* deletion under P_{help}
CΔ*trxA*-b	CGCGGATCCTTAAACGTATTTGTTGATGACTTCATCCAG		
CΔ*trxA*-c	CGCGAGCTCACAAGTGAGCAAAAACATGGCTCCA	408	Used for complementation of the *trxA* deletion under P_{trxA}
CΔ*trxA*-d	CCGCTCGAGTTAAACGTATTTGTTGATGACTTCATCCAG		
CΔ*lmo1059*-fwd	TTTCCATGGATATTAGTCAAATTAAAGCAGA	545	Used for complementation of the *lmo1059* deletion
CΔ*lmo1059*-rev	TCTGGATCCTTATTTAGCTAATTCATCATCAAGTAGC		

CΔ*lmo0964*-fwd	CGCGGATCCGATTAACCAAAATTTATATTATCAGTCAGTAGCAAAT	835	Used for complementation of the *lmo0964* deletion
CΔ*lmo0964*-rev	CCGCTCGAGTTATAAGTTTCCGATGTATTTCCAGTGAGA		
TrxA-exp-fwd	TTTCATATGGTAAAAGAAATTACAGATGCAACATTTG	324	Used for recombinant TrxA expression in *E. coli*
TrxA-exp-rev	CCGCTCGAGAACGTATTTGTTGATGACTTCATCCAGTTC		
TrxB-exp-fwd	TTTCATATGGCTAGTGAAGAAAAAATTTATGATGTG	972	Used for recombinant TrxB expression in *E. coli*
TrxB-exp-rev	CCGCTCGAGTTTAGCCGCCTCAGCTTCTAGC		
GmaR-exp-fwd	CGCCATATGCGGCCGTTAATTTCGATTTGTATG	1921	Used for recombinant GmaR expression in *E. coli*
GmaR-exp-rev	CCGCTCGAGTCGATTGTTTGTAACAGTGTCTTGAAGC		
MogR-exp-fwd	CGCCATATGCCTAAATCAGAAATAAGAAAATTACTTCAAGAAAT	928	Used for recombinant MogR expression in *E. coli*
MogR-exp-rev	CCGCTCGAGCATTTGTTTATAATTTTCTTTGAATACACCAAGT		
Lmo1059-exp-fwd	TTTCATATGGATATTAGTCAAATTAAAGCAGAAG	543	Used for recombinant Lmo1059 expression in *E. coli*
Lmo1059-exp-rev	CCGCTCGAGTTTAGCTAATTCATCATCAAGTAGCG		
Lmo0964-exp-fwd	GGAATTCCATATGATTAACCAAAATTTATATTATCAGTCAGTAGCAAATTC	835	Used for recombinant Lmo0964 expression in *E. coli*
Lmo0964-exp-rev	CCGCTCGAGTAAGTTTCCGATGTATTTCCAGTGAGACTG		
PrfA-exp-fwd	GGAATTCCATATGATGAACGCTCAAGCAGAAGAATTCA	730	Used for recombinant PrfA expression in *E. coli*
PrfA-exp-R	CCGCTCGAGATTTAATTTTCCCCAAGTAGCAGGACAT		
TrxA_C28A-fwd	CTGGGCAACATGGGCTGGTCCTTGCCGCATG	5547	Used for site-directed mutagenesis of TrxA
TrxA_C28A-rev	CATGCGGCAAGGACCAGCCCATGTTGCCCAG		
TrxA_C31A-fwd	GGCAACATGGTGTGGTCCTGCCCGCATGGTG	5547	
TrxA_C31A-rev	CACCATGCGGGCAGGACCACACCATGTTGCC		
TrxA_C28AC31A-fwd	GAGCCACCATGCGGGCAGGACCAGCCCATGTTGCCCAGA	5547	
TrxA_C28AC31A-rev	TCTGGGCAACATGGGCTGGTCCTGCCCGCATGGTGGCTC		

TrxA-EMSA-fwd	CGGTTCATAGTAGTGGTTCTCCAGCT	353	Used for amplifying the promoter region of *trxA*
TrxA-EMSA-rev	TTACCATGATTTAATTCACTCCTTATGACTT		
TrxB-EMSA-fwd	GATGCTGGGAAAGATTTAATACTTGGTCG	304	Used for amplifying the promoter region of *trxB*
TrxB-EMSA-rev	AAGCTGCTGTCATTCCAGCAGGC		
prfA-RT-fwd	TGCGGTCAACTTTTAATCCTG	112	Used for validating of the transcriptome results by RT-PCR
prfA-RT-rev	CGATGCCACTTGAATATCCTAACT		
plcA-RT-fwd	AGCCATTAGTCATTAACTCACGC	88	
plcA-RT-rev	ACACTCGGACCATTGTAGTCAT		
hly-RT-fwd	ACTGGTTTAGCTTGGGAATGG	107	
hly-RT-rev	TATTTCGGATAAAGCGTGGTG		
mpl-RT-fwd	GGAAGTTGCGAGTTGGATTGT	98	
mpl-RT-rev	TGTGGGTATCAGCCCGTTC		
actA-RT-fwd	GCCGAGCCTACCAGTAATCC	139	
actA-RT-rev	TTCAATGCCAGCAGAACGA		
plcB-RT-fwd	AAAGGTGGTTCTAGGTATGTGCTT	101	
plcB-RT-rev	GGAGCTGCGGGTGTTTGTA		
inlC-RT-fwd	ATTCAACGACCAACGCCTATT	94	
inlC-RT-rev	GGTCTGTAACACTTTGCTTCCCT		
lmo2185-RT-fwd	TCAACCACTTGCGGCTCAT	141	
lmo2185-RT-rev	GGAGGCGTTTCGTCACTACC		
lmo0485-RT-fwd	ATTACGCCGCCATACTTACTCAT	134	
*lmo048*5-RT-rev	ATCCCAATTCCCGCTTCC		
lmo2181-RT-fwd	ACAAAGGATGCCGCTACTACAA	96	
lmo2181-RT-rev	CGTCAGCCACCCAACCATAT		
lmo0362-RT-fwd	GGAGCCGCCCTTGTGATTT	98	
lmo0362-RT-rev	ATGCCTTTGGTCGCATTT		

lmo0361-RT-fwd	CATGAGCCTCACTGGACACC	84
lmo0361-RT-rev	CCCACAAAGAAAGCGAGAAA	
fliE-RT-fwd	ATGCAGCCAGCGTCTTACC	97
fliE-RT-rev	CTATCCAGCATTTGCGTGAAG	
lmo1961-RT-fwd	GGTCCCATTGGCCTATTCTC	83
lmo1961-RT-rev	CCGACAGCAGGTTCAGCAT	
lmo1516-RT-fwd	CTGGGAACGGCTTTATAGGTAG	91
lmo1516-RT-rev	TGTGGAATGGCGTCTGAGTAA	
lmo2390-RT-fwd	CAGGAATTTATTGTGCAGGTGA	97
lmo2390-RT-rev	GCATTATTGACGGCAGTTGGT	
sod2-RT-fwd	GAAGCGGTTGCTGGTCATC	81
sod2-RT-rev	ATCTTCAGGAACGCTATCTAGGTCTA	
flgG-RT-fwd	CCAGTTTCATTCCGATCAGTTT	70
flgG-RT-rev	TTGAATCGCTTCTCCAGTCG	
